# Differential CCR7 Targeting in Dendritic Cells by Three Naturally Occurring CC-Chemokines

**DOI:** 10.3389/fimmu.2016.00568

**Published:** 2016-12-09

**Authors:** Gertrud M. Hjortø, Olav Larsen, Anne Steen, Viktorija Daugvilaite, Christian Berg, Suzan Fares, Morten Hansen, Simi Ali, Mette M. Rosenkilde

**Affiliations:** ^1^Department of Neuroscience and Pharmacology, Faculty of Health and Medical Sciences, The Panum Institute, University of Copenhagen, Copenhagen, Denmark; ^2^Department of Haematology, Center for Cancer Immune Therapy (CCIT), Copenhagen University Hospital, Herlev, Denmark; ^3^Medical Faculty, Institute of Cellular Medicine, Newcastle University, Newcastle upon Tyne, UK

**Keywords:** CCR7, CCL19, CCL21, tailless-CCL21, dendritic cell, biased signaling, ERK

## Abstract

The CCR7 ligands CCL19 and CCL21 are increasingly recognized as functionally different (biased). Using mature human dendritic cells (DCs), we show that CCL19 is more potent than CCL21 in inducing 3D chemotaxis. Intriguingly, CCL21 induces prolonged and more efficient ERK1/2 activation compared with CCL19 and a C-terminal truncated (tailless) CCL21 in DCs. In contrast, tailless-CCL21 displays increased potency in DC chemotaxis compared with native CCL21. Using a CCL21-specific antibody, we show that CCL21, but not tailless-CCL21, accumulates at the cell surface. In addition, removal of sialic acid from the cell surface by neuraminidase treatment impairs ERK1/2 activation by CCL21, but not by CCL19 or tailless-CCL21. Using standard laboratory cell lines, we observe low potency of both CCL21 and tailless-CCL21 in G protein activation and β-arrestin recruitment compared with CCL19, indicating that the tail itself does not improve receptor interaction. Chemokines interact with their receptors in a stepwise manner with ultimate docking of their N-terminus into the main binding pocket. Employing site-directed mutagenesis we identify residues in this pocket of selective CCL21 importance. We also identify a molecular switch in the top of TM7 important for keeping CCR7 in an inactive conformation (Tyr312), as introduction of the chemokine receptor-conserved Glu (or Ala) induces high constitutive activity. Summarized, we show that the interaction of the tail of CCL21 with polysialic acid is needed for strong ERK signaling, whereas it impairs CCL21-mediated chemotaxis and has no impact on receptor docking consistent with the current model of chemokine:receptor interaction. This indicates that future selective pharmacological targeting of CCL19 versus CCL21 should focus on a differential targeting of the main receptor pocket, while selective targeting of tailless-CCL21 versus CCL21 and CCL19 requires targeting of the glycosaminoglycan (GAG) interaction.

## Introduction

Chemokines are chemotactic cytokines that are vital for the immune system, especially as mediators of immune cell recruitment to sites of inflammation. The chemokine receptors, mainly expressed on leukocytes, belong to the largest class of proteins in the human genome, namely, seven transmembrane (7TM) G protein-coupled receptors (also known as GPCRs). The chemokines are organized into groups according to the spacing between two conserved cysteine residues. This gives rise to four groups: CC, CXC, XC, and CX_3_C chemokines, with the number of Xs’ indicating the number of amino acids separating the conserved cysteines. The chemokine system is very promiscuous in that one ligand can bind to one or more receptors and *vice versa*. The chemokines CCL19 and CCL21 [previously known as EBI1-ligand chemokine (ELC) and secondary lymphoid tissue chemokine (SLC), respectively] are the only ligands for CCR7 (1–3); a receptor expressed on different subsets of immune cells (4), and involved in the homing of naive T cells and antigen-presenting dendritic cells (DCs) to the lymph nodes. In the lymph nodes, DC–T cell priming takes place to allow antigen-specific T-cell activation (5). In addition to its role in protective immunity, the CCR7:CCL19/CCL21 axis is also believed to be important for the architecture of the thymus (6, 7) and for the induction of peripheral tolerance (5, 8).

Although CCL19 and CCL21 bind to the same receptor, they are differentially expressed; i.e., CCL19 is secreted by mature DCs, whereas CCL21 is secreted from the endothelium of afferent lymphatic vessels, and they both are present in the lumen of high endothelial venules (HEVs) and in stromal cells of the lymph node (9–11). CCL19 and CCL21 only share 32% amino acid identity, and importantly CCL21 has a large C-terminal tail of 37 amino acids that is highly positively charged and capable of strong binding to glycosaminoglycans (GAGs) causing chemokine immobilization (12, 13). CCL19 lacks this C-terminal domain and has poor affinity for GAGs. Due to their differential expression pattern and structural differences, it was early speculated that CCL19 and CCL21 binding to CCR7 would induce distinct cellular responses (9–12). Moreover, it was recently shown that the tail of CCL21 causes the chemokine to adopt an auto-inhibited conformation in the absence of polysialic acid interaction (14). Finally, a tailless version of CCL21 was recently shown to be generated naturally through cleavage of full-length CCL21 initiated by endogenous proteases released by DCs (15), indicating that so far, three configurations of CCR7 activity can be anticipated in this cell type.

The canonical signaling for GPCRs is *via* coupling to heterotrimeric G proteins, which in turn are activated and initiate downstream signaling pathways. However, in the recent years, it has been shown that intracellular signaling also occurs following β-arrestin-mediated internalization (16). The knowledge of diversity in signaling pathways, has opened up for the concept of *biased signaling*, which involves a preference for one signaling pathway over another, e.g., G protein versus β-arrestin coupling. It can be considered as either receptor bias (where the same ligand has different actions through different receptors), ligand bias (where two or more ligands act on the same receptor and induce different outcomes), or tissue bias (where the cellular effect depends on the tissue/cell type) (17, 18). Ligand bias by CCL19 and CCL21 at CCR7 has previously been described with regard to G protein coupling, β-arrestin recruitment, and receptor internalization in various stably transfected cell lines (19–22). In addition, differential tissue expression of CCL19 and CCL21 along with GAG accumulation of CCL21, polysialic acid control of full-length CCL21 activity, and local enzymatic cleavage of CCL21 to generate tailless-CCL21, is expected to confer tissue bias to CCR7.

For future therapeutic initiatives, it is important to understand the roles of CCL19 and CCL21 in shaping immune cell migration and activation to potentially separate good and bad effects of CCR7 activity and target it accordingly. Here we investigate CCL19 and CCL21 for their ability to induce chemotaxis of human monocyte-derived DCs. As migration has classically been linked to ERK activation, we investigate the effect of CCL19 and CCL21 on CCR7 signaling through this pathway in DCs and assess the influence of sialic acid residues on ERK activity and migration. To delineate a possible role of the elongated C-terminal basic tail of CCL21 in CCR7 signaling, we evaluate the potency of a tailless version of CCL21 in inducing DC migration and ERK activation, and also assess CCL19, CCL21, and tailless-CCL21 for their abilities to differentially activate CCR7 with respect to various intracellular effectors, including G protein activation, β-arrestin recruitment, and receptor internalization. Finally, through a mutational scan, we search for residues in CCR7 of differential importance for CCL19 or CCL21 in order to determine structural differences controlling receptor docking of their respective N-termini into the main binding pocket of CCR7.

## Materials and Methods

### Materials

The human chemokines CCL19 and CCL21 were purchased from R&D systems and PeproTech (Accession # Q99731.1 and Q6ICR7, respectively). Tailless-CCL21 was from ALMAC (sequence SDGGAQDCCL KYSQRKIPAK VVRSYRKQEP SLGCSIPAIL FLPRKRSQAE LCADPKELWV QQLMQHLDKT PSPQKPAQG). Goat anti-human CCL21 was from R&D systems (Cat. no. AF366). The human CCR7 cDNA was cloned from a spleen-derived cDNA library. The promiscuous chimeric G protein Gα_Δ6qi4myr_ (G_qi4myr_) that converts Gα_i_-related signaling into a Gα_q_ readout (23, 24) was kindly provided by Evi Kostenis (University of Bonn, Germany). Antibodies against p44/42 MAPK (ERK1/2) and phosphorylated p44/42 MAPK (ERK1/2, Thr202/Tyr204) were purchased at Cell Signaling. Neuraminidase (NA) was from Sigma.

### Transfections and Cell Culture

HEK293 cells were grown at 10% CO_2_ and 37°C in Dulbecco’s modified Eagle’s medium 1885 supplemented with 10% fetal bovine serum, 2 mM glutamine, 180 U/ml penicillin, and 45 μg/ml streptomycin. PathHunter U2OS β-Arrestin 2 Parental cell line (DiscoveRx, Birmingham, United Kingdom) were grown at 5% CO_2_ and 37°C in MEMα Glutamax medium supplemented with 10% fetal bovine serum, 180 U/ml penicillin, 45 μg/ml streptomycin, and 0.25 μg/ml Hygromycin B (Invitrogen). Transient transfection of HEK293 cells for phosphatidylinositol (PI)-turnover was performed using the calcium phosphate precipitation method as previously described (25, 26). U2OS cells were transfected using FuGENE^®^ 6 Transfection reagent (Roche, Mannheim, Germany).

### Phosphatidylinositol-Turnover

HEK293 cells were cotransfected with receptor cDNA and G_qi4myr_, which converts the Gα_i_ signal into a Gα_q_ signal, making it possible to measure the chemokine receptor activation as PI-turnover (23, 24). One day after transfection, the cells were seeded in 96-well plates (3.5 × 10^4^ cells/well) and incubated with 0.65 μCi of ^3^H-*myo*-Inositol in 0.1 ml growth medium for 24 h. Cells were washed twice with Hank’s buffered salt solution (HBSS) supplemented with CaCl_2_ and MgCl_2_ and incubated for 15 min in 0.1 ml buffer supplemented with 10 mM LiCl prior to ligand addition followed by 90 min incubation. The generated [^3^H]inositol phosphate was detected directly by addition of SPA-YSI bead solution. Determinations were made in duplicates.

### β-Arrestin Recruitment Assay

Recruitment of β-arrestin was measured using the PathHunter™ β-arrestin assay (DiscoveRx). CCR7 was fused with the ProLink™ (PK) 1-tag [a small fragment of the enzyme β-galactosidase (β-gal), the enzyme donor] and cloned into a pCMV-vector. Assays were performed in a U2OS cell line stably expressing β-arrestin 2 coupled to the large β-gal fragment (enzyme acceptor). Cells were seeded in 96-well plates, 20,000 cells/well, and transfected the following day with 50 ng DNA using FuGENE^®^ 6 reagent (0.15 μl/well). Twenty-four hours after transfection, the medium was removed and 100 μl Opti-MEM^®^ I (Gibco^®^) was added. The following day, cells were stimulated with varying concentrations of ligand for 90 min at 37°C. The Detection Reagent Solution^®^ was added prior to 60 min incubation at room temperature and the β-arrestin recruitment was measured as chemiluminescence using Perkin Elmer EnVision 2104 Multilabel Reader.

### Internalization

The internalization of CCR7 was measured in the U2OS cell line from DiscoveRx. The cells coexpressed enzyme acceptor-tagged β-arrestin 2 and a PK-tag linked to endofin, which is localized to endosomes. The experiment was carried as described for the β-arrestin recruitment assay, yet with 3 h incubation with the ligands.

### DC Maturation

Monocyte-derived DCs (DCs) were prepared by plastic adhesion from peripheral blood mononuclear cells (PBMCs) kindly provided by Professor Inge Marie Svane, Herlev Hospital, Denmark. The monocytes were differentiated into immature DCs in X-Vivo medium supplemented with 1000 U/ml GM-CSF and 250 U/ml IL-4 for 6 days, followed by maturation for 2 days with 1000 U/ml TNF-α, IL-1β, and IL-6 plus 1 μg/ml PGE_2_. Harvest of DCs was performed by medium aspiration followed by cold incubation of remaining cells in PBS with EDTA (5 mM) and subsequent scraping of cells followed by freezing of DCs in aliquots.

### Measurement of ERK Phosphorylation (Mesoscale Multisport Assay)

Mature DCs were seeded in 96-well plates (8.0 × 10^4^ cells/well). DCs were incubated for 2 h at 37°C, 5% CO_2_ before addition of chemokine and incubation at 37°C for 10 min. Following, the cells were lysed and prepared for measurement of ERK1/2 phosphorylation by using the Meso Scale Discovery (MSD)^®^ MULTI-SPOT Assay system Phospho (Thr202/Tyr204; Thr185/Tyr187)/Total ERK1/2 Assay (Meso Scale Discovery, MD, USA) according to the manufacturer’s instructions. Determinations were made in duplicates and measurements were performed on MSD SECTOR Imager.

### Measurement of ERK Phosphorylation (Western Blot)

Mature DCs were washed twice in serum-free X-vivo medium and incubated in this medium for 2 h at RT to starve them from serum factors. In experiments with NA treatment, the enzyme was added during this incubation at a concentration of 2.5 × 10^−2^ U/ml and the cells kept at 5% CO_2_ and 37°C. The cells were seeded in 96-well plates (8 × 10^4^ cells/well) and stimulated with CCL19, CCL21, or tailless-CCL21 for the indicated time period. The cells were lysed in ice-cold RIPA buffer (Millipore) with protease (mini complete Roche) and phosphatase inhibitors (mix 2 and 3, Sigma). The lysates were run on SDS-gel and transferred to PVDF for detection of total and phosphorylated ERK1/2.

### Ibidi^®^ 3D Migration Assay

The Ibidi^®^ assay was carried out according to the manufacturer. Briefly, the collagen mixture was prepared and DCs were added to a final concentration of 0.5 to 1 × 10^6^ cells/ml. The cell/collagen mixture was loaded into the Ibidi channel, according to the protocol, and left to polymerize for 35 min in a 5% CO_2_, humidified incubator at 37°C. The final collagen concentration was 1.67 mg/ml. Finally the source and sink reservoirs were filled with medium containing 2% human serum with or without chemokine, respectively, and the slide with loaded DCs was mounted on a computer controlled stage, holding a temperature controlled (37°C), humidified incubation chamber with 5% CO_2_. DC migration was followed for 12 h by time-lapse microscopy using a time interval of 2 min. Cell migration (approximately 20–40 cells) was tracked using a commercial tracking program (Autozell) and subsequently analyzed to get a population-based chemotactic index (CI) value (MATLAB). CI is a measure of net translocation distance to the source relative to total distance traveled.

### Immunostainings

In order to determine ligand surface localization, DCs were stained and fixed in suspension by incubating in serum-free medium with or without 100 nM CCL21, or tailless-CCL21 for 30 min on ice, followed by washing in PBS and fixation for 15 min in 3.7% formaldehyde in PBS. After fixation the cells were washed and incubated for 1 h with primary anti-human CCL21 antibody. The DCs were washed and incubated with secondary rabbit anti-goat antibody (Alexa-488 coupled) for 1 h. The DCs were washed, in PBS, with an additional final wash in sterile water and resuspended in 70% ethanol. DCs were mounted on microscope slides and sealed under coverslides.

### ELISA

CCL21 and tailless-CCL21 were adsorped on Maxisorp plates in PBS buffer over night at 4°C. The wells were washed three times in PBS and incubated for 60 min in blocking buffer (PBS, 1% BSA) at room temperature. Wells were incubated for 60 min with primary goat anti-CCL21 antibodies in blocking buffer. After PBS washing, the wells were incubated with secondary rabbit anti-goat HRP antibodies for 60 min. After PBS washing, TMB substrate was added. The reaction was stopped with H_2_SO_4_ 0.2M and absorbance at 450 was read in an Envision plate reader.

### Calculations

IC_50_, EC_50_, and *K*_d_/*K*_i_ values were determined by non-linear regression and *B*_max_ values were calculated using the GraphPad Prism 4.0 software (GraphPad, San Diego, CA, USA).

### Statistical Analysis

Statistical significance was calculated using unpaired *t* test, and data are represented as mean ± SEM. Significance is indicated as follows: NS, not significant; *P < 0.05; **P < 0.01.

## Results

### Decreased Response to CCL21 in 3D Chemotaxis of Human DCs

**Figure 1:**
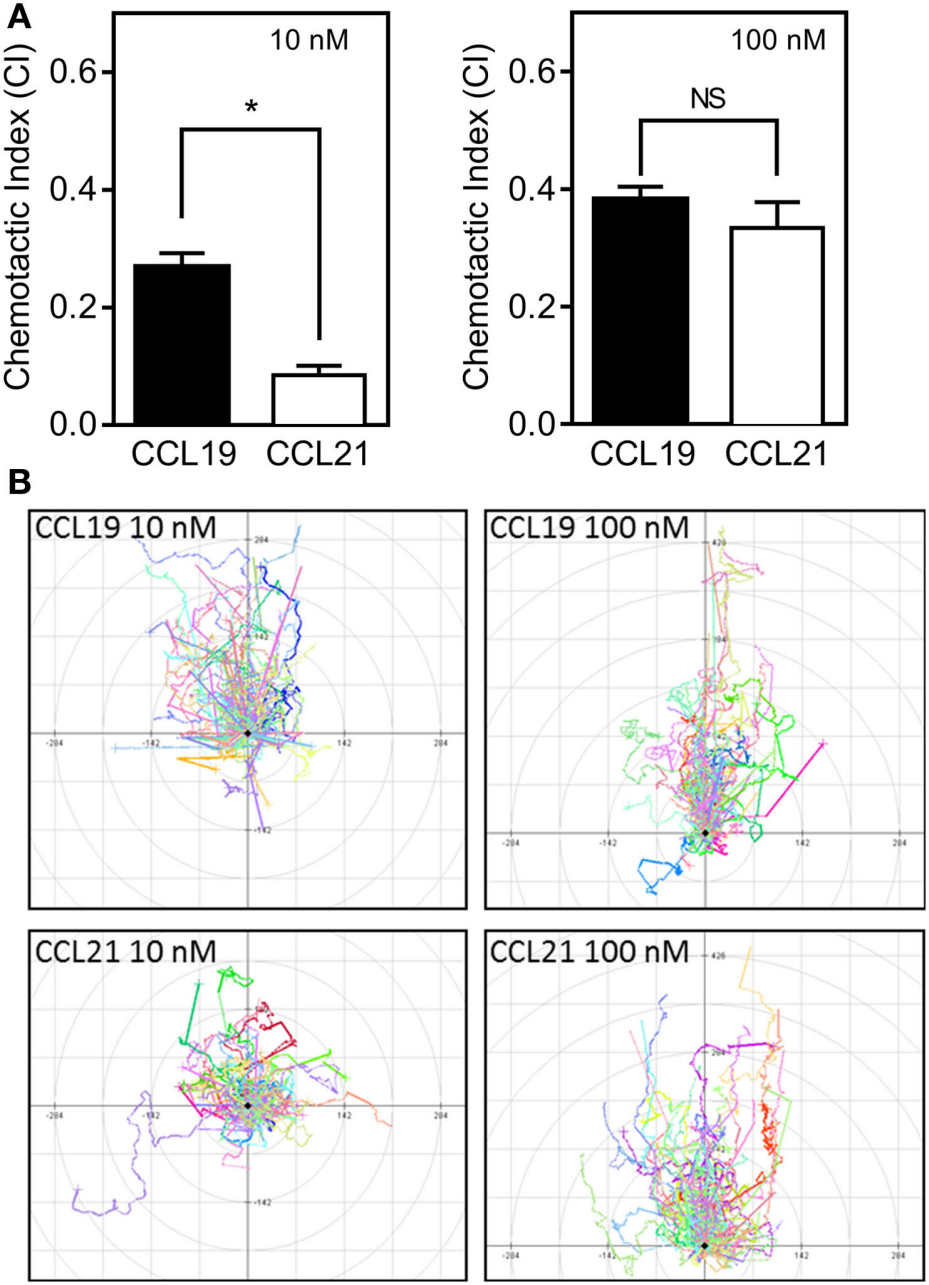
**3D chemotaxis of human dendritic cells (DCs) in response to CCL19 and CCL21**. Human DCs naturally expressing CCR7 were used for the 3D chemotaxis assay. In this assay, a collagen filled channel with the cells is in contact with a source and sink reservoir on either side, causing the cells to experience a linear gradient as chemokine gradually diffuses from source to sink. **(A)** Column diagrams showing the directional migration of DCs in response to either 10 nM (left) or 100 nM (right) CCL19 or CCL21 source concentrations. The values are calculated as chemotactic index (CI) in the MATLAB software. **(B)** Spider diagrams depicting the migration pattern in response to the indicated concentrations of the chemokines. Statistical significance was calculated using unpaired *t* test. NS, not significant; *P < 0.05 (*n* = 3).

To assess the relative importance of the two CCR7 ligands in stimulating various DC responses, we investigated the effect of CCL19 and CCL21 on a range of biological readouts. One very important readout in immune cell biology is the ligand-controlled migration of cells toward physiologically relevant ligands. We measured chemotaxis of human monocyte-derived DCs induced by either CCL19 or CCL21 using Ibidi^®^ 3D chemotaxis slides. At 10 nM, CCL21 induced a migration response characterized by a significantly lower CI compared with CCL19. However, at 100 nM chemokine concentrations, the cells migrated with equal directionality (CI) toward the two ligands (Figure 1A). The migration patterns are depicted in the spider diagrams (Figure 1B), which show that DC migration is less directional in response to CCL21 compared with CCL19 at low concentrations (10 nM).

### Superior MAP Kinase Activation by CCL21 Compared with CCL19 in Human DCs

**Figure 2:**
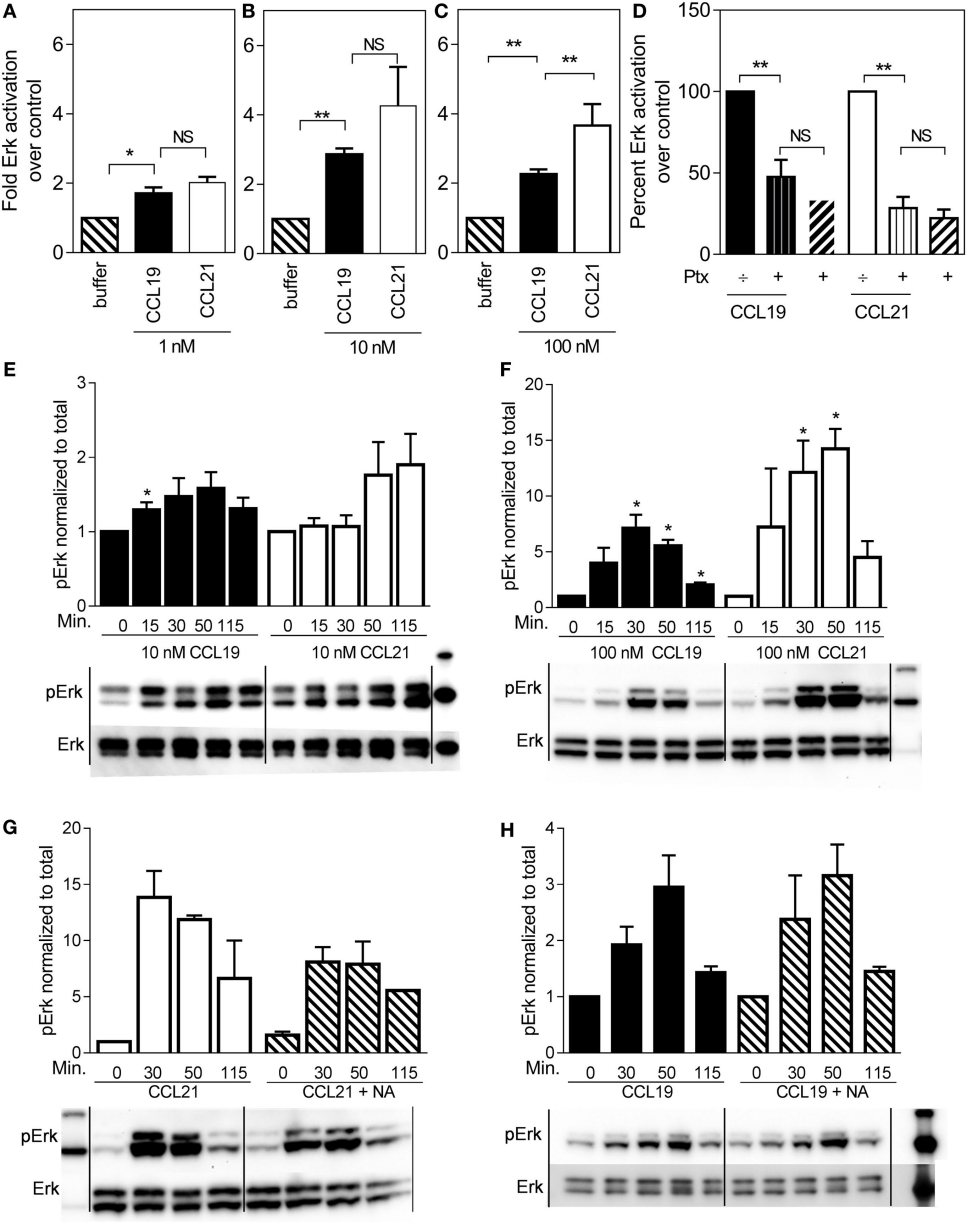
**ERK1/2 activation in human dendritic cells in response to CCL19 and CCL21**. Human DCs with natural CCR7 expression were used for the ERK1/2 activation. The percentage of phosphorylated ERK1/2 was calculated as described by the manufacturer (Meso Scale Discovery, MD, USA) as % Phosphoprotein = [(2 × Phospho-signal)/(Phospho-signal + Total signal)] × 100. The pERK1/2 data are normalized to buffer and displayed as fold ERK1/2 activation over buffer control. **(A–C)** The effect of 1 nM **(A)**, 10 nM **(B)**, or 100 nM **(C)** of CCL19 and CCL21. **(D)** The effect of PTX (10 μg/ml) on ERK1/2 activation by CCL19 and CCL21. **(E)** Timely effect of 10 nM and **(F)** 100 nM CCL19 and CCL21 on DC ERK1/2 phosphorylation. **(G)** The effect of neuraminidase (NA) treatment of DCs on ERK1/2 activation by CCL21 and **(H)** by CCL19. Statistical significance was calculated using unpaired *t* test. NS, not significant; *P < 0.05, **P < 0.01 (*n* = 3–6).

ERK is often associated with migration, and therefore we tested ERK activation profiles in mature human DCs stimulated with either CCL19 or CCL21. Surprisingly, CCL21 induced more ERK activity than CCL19 (Figure 2). At 100 nM, CCL21 induced an approximately fourfold increase in pERK1/2 over cells stimulated with buffer, whereas the same amount of CCL19 only induced an approximately twofold increase (Figure 2C). The same tendency was seen at 1 and 10 nM chemokine concentrations (Figures 2A,B).

In order to determine G protein contribution to the MAP kinase activity, ERK activity was measured in the presence of pertussis toxin (PTx), a substance that inhibits Gα_i_ irreversibly by ADP-ribosylation. As seen in Figure 2D, 10 μg/ml PTx completely abolished CCL19- and CCL21-induced ERK1/2 activation in DCs, indicating that Gα_i_ is the main mediator of CCR7-induced pERK1/2 activation in DCs.

Due to the established broad spectrum GAG binding of CCL21 through its C-terminal tail (12, 13), a local CCL21 reservoir is expected to form at the DC surface, which may gradually become available for CCR7 activation through various mechanisms, influencing the CCL21 ERK activation profile over time. Thus, we investigated time-dependent MAP kinase activation. At 10 nM chemokine concentrations, there was a tendency that CCL19-induced ERK activation at an earlier time-point than CCL21, which shows a tendency to peak later (Figure 2E). At 100 nM chemokine concentrations, ERK activation was in general more pronounced. ERK activation by CCL21 was prolonged and peaked at 50 min after induction (or even later), whereas that of CCL19 peaked earlier – at 30 min (or possibly before) (Figure 2F). The stronger CCL21-induced ERK activation giving rise to the hypothesis of CCL21 forming a local reservoir.

The GAG-subtype polysialic acid was recently shown to act as coreceptor for CCL21 during CCR7 activation (14), whereas others, e.g., chondroitin sulfate-B (CS-B) have been shown to inhibit CCL21 activity. To determine the impact of polysialic acid on CCL21-induced ERK activation, DCs were pretreated with NA that was also present during the experiment. Indeed, this treatment negatively affected CCL21-induced ERK1/2 activation (Figure 2G), but not CCL19-induced activity (Figure 2H), supporting an important role of polysialic acid in CCL21-induced CCR7 activation and thus ERK signaling in DCs.

### Tailless-CCL21 Is Superior to CCL21 in Inducing DC Migration and Also Resembles CCL19 with Regard to ERK Activation

Since the release of the C-terminal tail of CCL21 from its polysialic acid interaction negatively affects CCL21-mediated ERK activation (Figure 2G), we wanted to investigate the role of the extended C-terminal tail of CCL21 in chemotaxis and therefore designed a truncated version of CCL21 lacking the C-terminus and investigated its properties in inducing 3D migration and ERK activation of human DCs.

**Figure 3:**
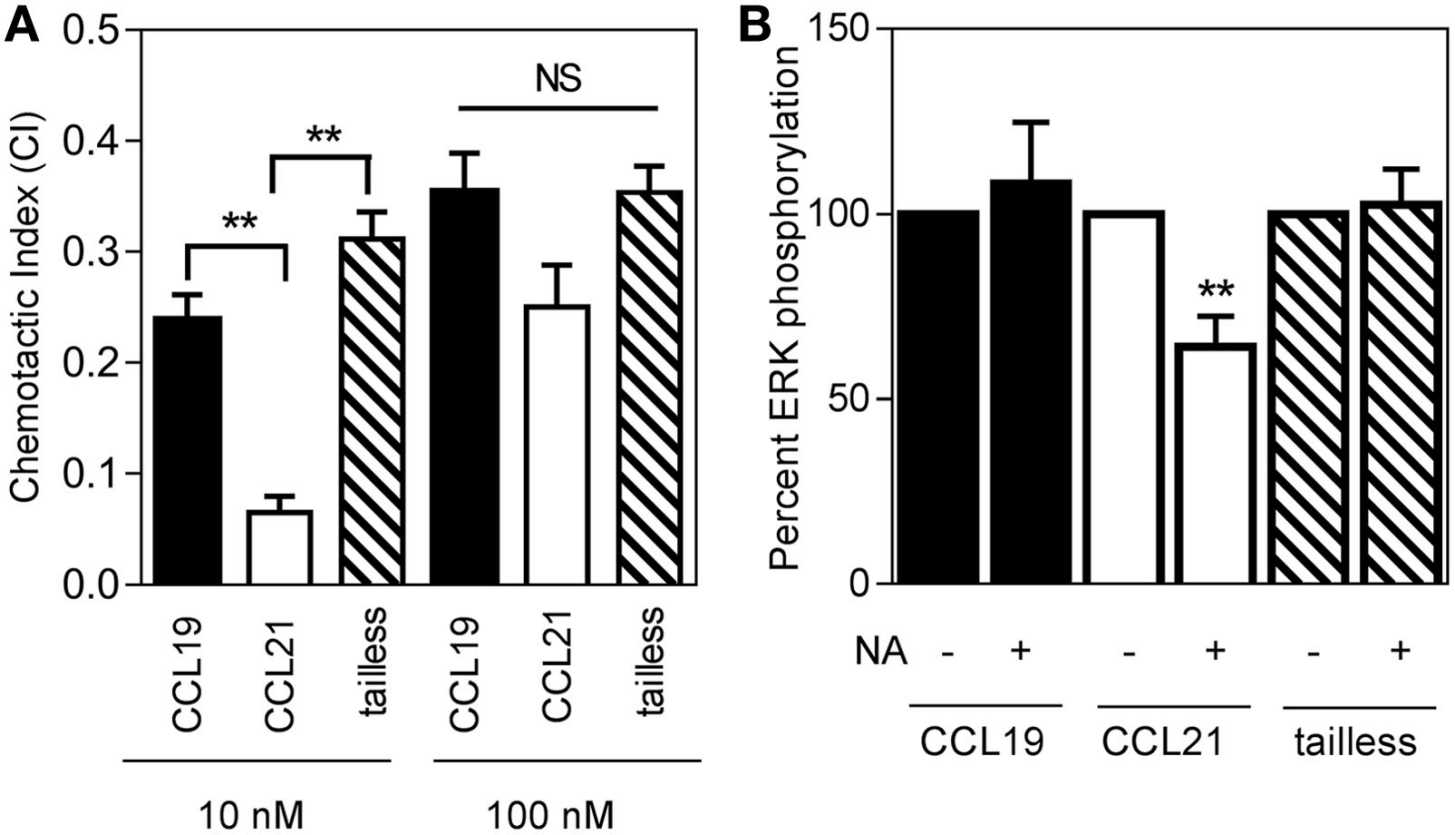
**Response of the three naturally occurring chemokines (CCL19, CCL21, and tailless-CCL21) on 3D chemotaxis of human dendritic cells**. **(A)** Column diagrams showing the directional migration of DCs in response to 10 nM and 100 nM of CCL19, CCL21, or tailless-CCL21 source concentrations. The values are calculated as chemotactic index (CI) in the MATLAB software. **(B)** Column diagrams showing the pERK in response to 100 nM CCL19, CCL21, or tailless-CCL21 in neuraminidase treated DCs as percentage of the signal of the corresponding chemokine in non-neuraminidase treated cells. Data are normalized, and all non-treated responses set to 100%. Statistical significance was calculated using unpaired *t* test. **P < 0.01, *P < 0.05, NS, not significant (*n* = 3).

Indeed, tailless-CCL21 was more potent than full-length CCL21 in inducing directed migration of DCs and at a concentration of 10 nM induced chemotaxis with similar CI as CCL19 (Figure 3A), whereas there was no significant difference between the three chemokines at 100 nM (Figure 3A). Tailless-CCL21 also resembled CCL19 with regard to ERK activation and thus in contrast to what was observed for CCL21, neither CCL19 nor tailless-CCL21-induced ERK activation was affected by DC NA treatment (Figure 3B).

### CCL21 Binds to the DC Surface Forming Large and Small Puncta

**Figure 4:**
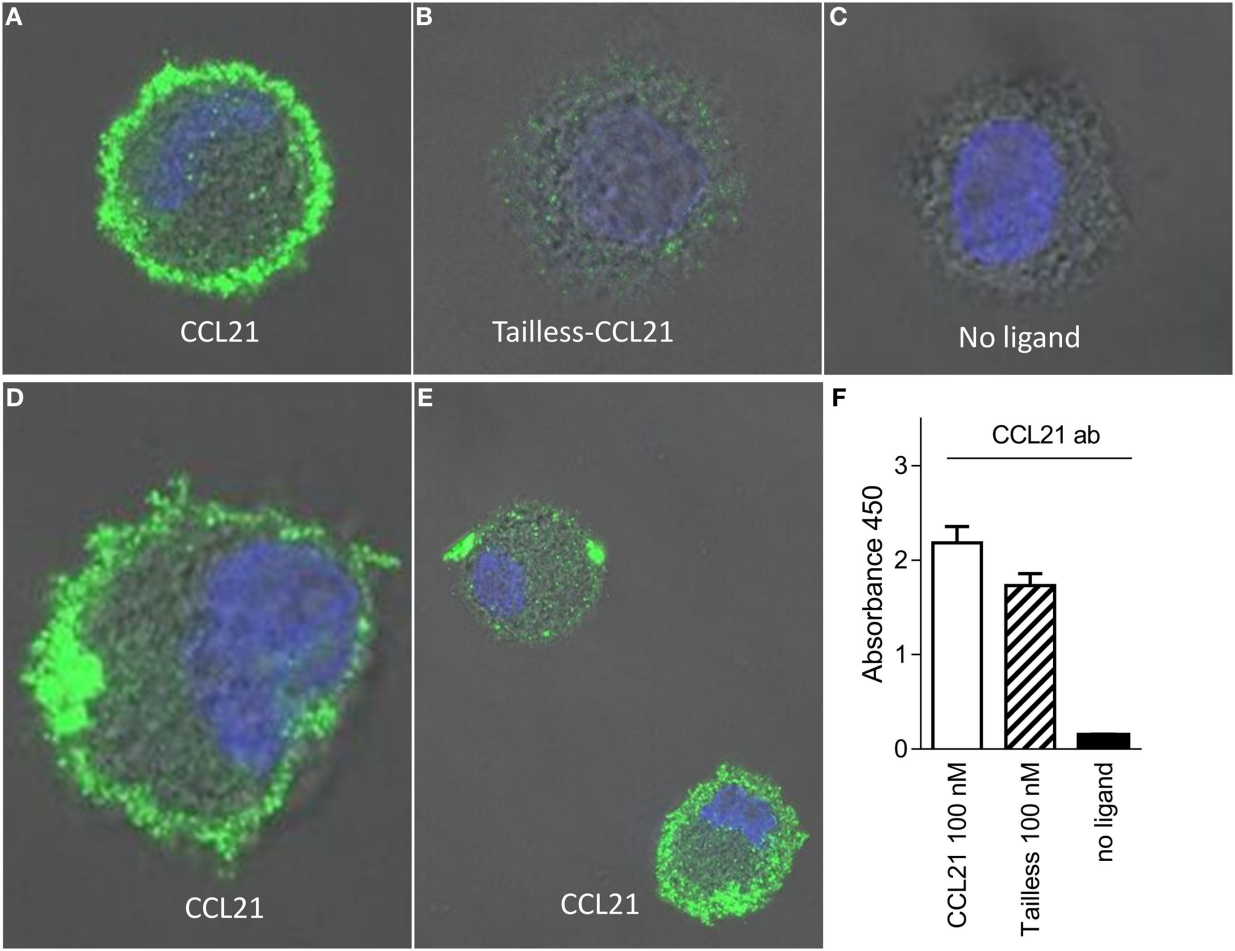
**CCL21 binds to the surface of human DCs and forms discrete puncta; a feature not matched by tailless-CCL21**. Fluorescence microscopy pictures of DCs incubated **(A)** with100 nM CCL21, **(B)** 100 nM tailless-CCL21, or **(C)** in the absence of ligand obtained on LSM 780 confocal microscope using 63× oil-objective (ligands were stained with Alexa 488 and the cell nucleus visualized with Hoecst DNA staining). **(D)** Zoom in on DC incubated with 100 nM CCL21 to visualize that anti-CCL21 staining followed the villi-like surface of non-adherent DCs. **(E)** Visualization of two different types of CCL21 puncta. **(F)** The anti-CCL21 antibody also recognized tailless-CCL21.

To test if CCL21 could be detected on the DC surface to a higher degree than tailless-CCL21, we performed immunostainings against the two chemokines on cells that had been incubated on ice with 100 nM of the respective chemokines. In contrast to tailless-CCL21, CCL21 localized to the DC membrane forming discrete puncta, whereas cells incubated in the absence of ligand were devoid of staining (Figures 4A–E). Membrane localization appeared as an extended rim around the cell that was not sharply defined due to the dendrites that make the membrane villi-like in appearance in non-adherent DCs (Figure 4D). CCL21 stainings in general took form of either a few large puncta or multiple small puncta, with both types of stainings following the rim of the cell (Figure 4E). Importantly, polyclonal antibody against CCL21 recognized tailless-CCL21 to the same degree as it recognized CCL21 (Figure 4F).

### CCL21 and Tailless-CCL21 Are Less Potent Compared with CCL19 in G Protein-Mediated Signaling and β-Arrestin Recruitment

Intrigued by the observations that tailless-CCL21 resembled CCL19 more than it resembled CCL21 in both migration and ERK activation, we investigated the potency of CCL19, CCL21, and tailless-CCL21 in inducing signaling pathways “closer” to the membrane (G protein activation, β-arrestin recruitment, and CCR7 internalization) as compared to the more downstream MAP kinase activation and migration.

The ability to induce signaling *via* Gα_i_ – the major G protein pathway induced by endogenous chemokine receptors (27) – was investigated in a PI-turnover assay using HEK293 cells transfected with CCR7 and the chimeric protein G_qi4myr_ that converts a Gα_i_-coupled response into a Gα_q_ readout (24).

**Figure 5:**
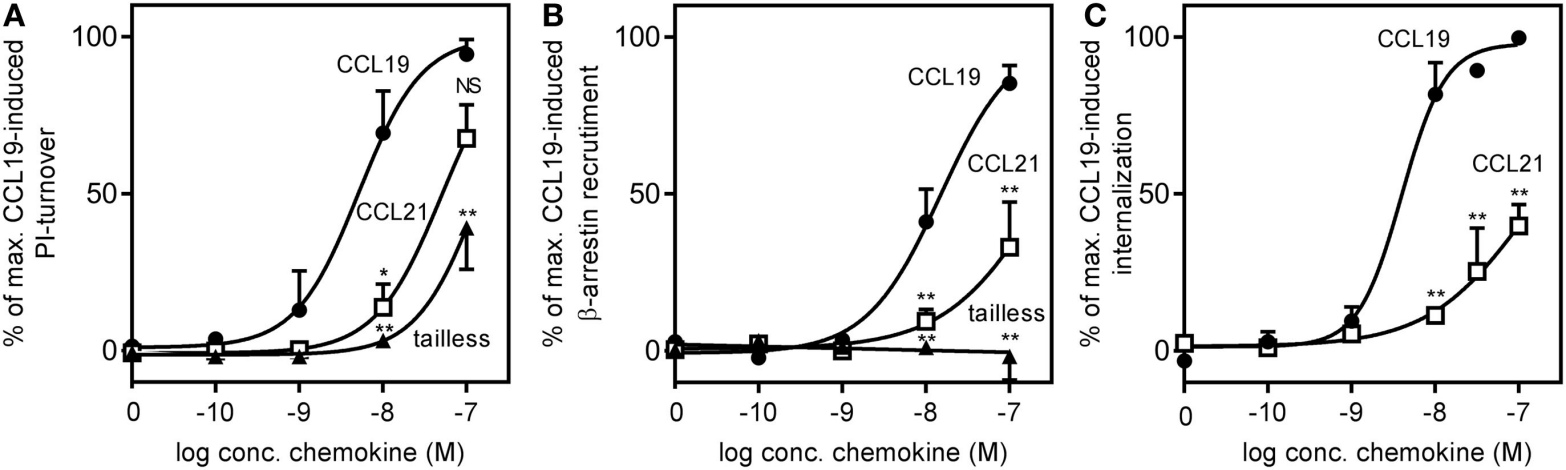
**CCR7 G protein activation, β-arrestin recruitment, and internalization induced by CCL19, CCL21, and tailless-CCL21**. **(A)** Dose–response curves of CCL19 (circles), CCL21 (squares), and tailless-CCL21 (triangles) obtained in PI-turnover assay measured in HEK293 cells cotransfected with CCR7 and the chimeric G protein G_qi4myr_. Statistical significant difference from CCL19 was calculated by unpaired Student’s *t*-test. *P < 0.05, **P < 0.01, and NS, not significant. **(B)** β-arrestin recruitment measured in U2OS cells stably transfected with β-arrestin 2 linked to an enzyme acceptor and transiently transfected with enzyme donor-fused CCR7 with increasing concentrations of CCL19 (circles), CCL21 (squares), and tailless-CCL21 (triangles). Statistical significance between CCL19 and CCL21 values was calculated using unpaired *t* test. **P < 0.01. **(C)** β-Arrestin 2-dependent internalization measured in U2OS cells coexpressing untagged CCR7 DNA, β-arrestin 2 fused to the enzyme acceptor part of β-gal, and endosomes tagged with PK, the enzyme donor. Dose–response curves for CCL19 (circles) and CCL21 (squares) are shown. Statistical significance was calculated using unpaired *t* test (*n* = 3–4).

We found that the potency of CCL21 was ~10-fold lower compared with that of CCL19 with an estimated EC_50_ of 50 nM (−log 7.3 ± 0.18, *n* = 3) for CCL21 compared with 5 nM for CCL19 (−log 8.3 ± 0.27, *n* = 3), and with no significant difference in efficacy between the two (Figure 5A). Surprisingly, in contrast to what was observed in migration (Figure 3A), tailless-CCL21 did not exhibit increased ability to signal *via* Gα_i_ but displayed low activity in this pathway similar to full-length CCL21 (estimated EC_50_ of 142 nM).

To determine receptor mediated β-arrestin recruitment, CCR7 tagged at the C-terminus with the catalytic N-terminal domain of β-gal, also referred to as the enzyme donor fragment, was transiently transfected in U2OS cells stably expressing β-arrestin 2 fused to an N-terminal deletion mutant of β-gal, the enzyme acceptor fragment. The binding of β-arrestin 2 to CCR7 reconstitutes donor and acceptor parts of β-gal into a functional enzyme and the readout was measured as chemiluminescence. As with Gα_i_ signaling, CCL19 exhibited a significantly higher potency compared with CCL21 (~17-fold), and CCL21 only reached half of CCL19-induced activity at 100 nM (Figure 5B). EC_50_ was estimated to 15 nM (−log 7.8 ± 0.1, *n* = 3) for CCL19 and 263 nM (−log 6.6 ± 0.32, *n* = 3) for CCL21. Again, tailless-CCL21 was weak; in fact it did not induce β-arrestin 2 recruitment at concentrations up to 100 nM. β-arrestin-mediated CCR7 internalization induced by CCL19 and CCL21 was measured in U2OS cells coexpressing untagged receptor DNA, β-arrestin 2 fused to the β-gal enzyme acceptor fragment, and endosomes tagged with β-gal enzyme donor fragment. Tailless-CCL21 internalization was not investigated due to lack of β-arrestin coupling. Quantitative fusion of β-arrestin and endosomes was detected as chemiluminescence. CCL21 induced internalization; however, only to ~40% of the level induced by CCL19 at 100 nM (Figure 5C). Its potency was also lower (~37-fold) with an EC_50_ estimated to 152 nM (−log 6.8 ± 0.08, *n* = 3) compared with 4.1 nM for CCL19 (−log 8.4 ± 0.14, *n* = 3).

Thus CCL21 is weaker than CCL19 in G protein activation and β-arrestin recruitment, and tailless-CCL21 resembles CCL21 more than CCL19 although it seems to be less potent compared with CCL21.

### CCL19 and CCL21 Diverge in Structure

**Figure 6:**
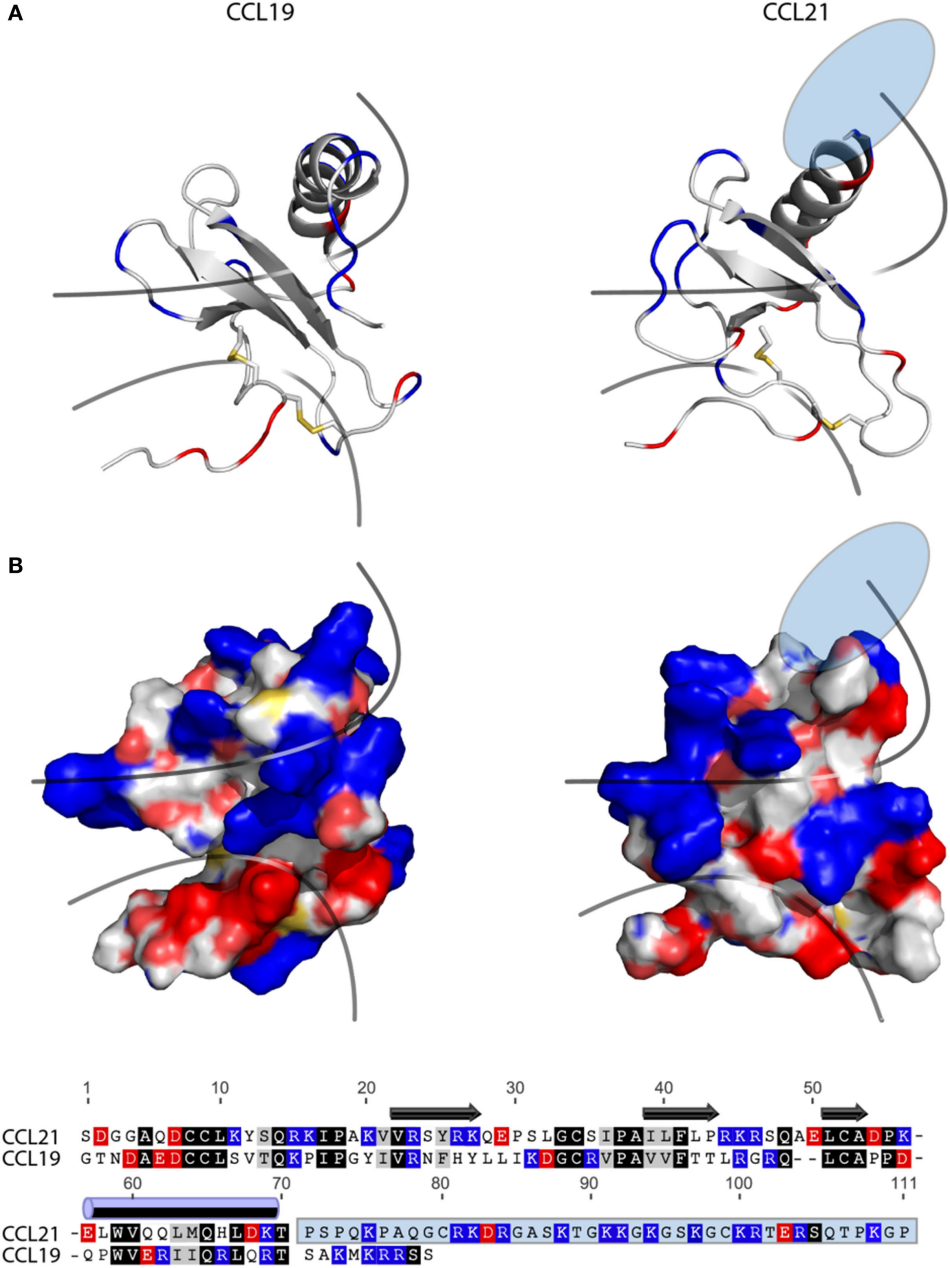
**Structure and alignment of CCL19 and CCL21**. **(A)** NMR solution structure of CCL21 (PDB reference 2L4N) and CCL19 (PDB reference 2MP1). Cartoon structures are aligned in PyMOL to demonstrate the secondary structures likely to be found in both proteins (top). Exposed charges of either structure are shown on the surface presentation (bottom) with positive charges in blue and negative charges in red. Pale blue and pale red represents surface-exposed nitrogen- and oxygen atoms, respectively. The lower line divides the chemokine core domain from the highly flexible N-terminus, whereas the upper line delimits the regions that most closely match the mark of a GAG-binding domain, i.e., a dense cluster of exposed positive charges. The light blue ellipse on CCL21 represents the large unsolved C-terminal tail, of which most is also removed in tailless-CCL21. **(B)** Amino acid sequence alignment of CCL19 and CCL21 using the MAFFT multiple-aligner plug-in of Geneious Pro 6.1.7 software (Biomatters Ltd., Auckland, New Zealand). The secondary structures are shown with symbols, using arrows for beta-strands and a cylinder for the alpha-helix. Identical residues are black, similar residues are gray, and positively and negatively charged residues are blue and red, respectively. The unsolved C-terminus of CCL21 is outlined in a light blue box.

The fact that tailless-CCL21 displays distinct properties compared with CCL19, and resembles CCL21 more with regard to upstream signaling events, implies that the tail of CCL21 plays no major role in controlling differences in CCL19 and CCL21 receptor engagement. In Figure 6A, a backbone solution structure (top) and a surface view (bottom) is presented of the NMR structure of CCL19 and CCL21, excluding the flexible C-terminal tail of CCL21 (28, 29). Considering overall structural and functional similarities between CCL19 and CCL21 (19–22), we compared the tertiary structures of these two chemokines. As the solution structure reveals, CCL21 maintains the triple-stranded beta-sheet and C-terminal alpha-helix typical to chemokines. The surface presentations underscore obvious differences between CCL19 and CCL21 in GAG but also in receptor docking domains. CCL21 has a much more pronounced clustering of positively charged amino acids in the GAG-binding domain, which probably facilitates the highly increased GAG-binding capability of this chemokine residing in the positively charged C-terminus extending directly from this region (Figure 6A), a feature not matched in CCL19. The primary sequence alignment (Figure 6B) highlights similarities and charges of CCL19 and CCL21. The highly basic GAG-binding tail of CCL21 clearly underscores the charge difference between CCL19 and CCL21 located to the GAG-binding domain.

It is generally believed that receptor activation occurs as a result of the N-terminal chemokine domain docking deep into the main binding pocket; a process thought to be a major contributor to the ligand-specific receptor activation mechanism (30, 31). CCL19 has a slightly more negative and relatively smaller (721 versus 649 Da) receptor docking domain compared with CCL21. Thus differences in size and distribution of electronegativity in the N-terminal chemokine domain probably dictate different receptor docking modes for CCL19 and CCL21.

### CCL19 and CCL21 Rely on Different Amino Acids for CCR7 Engagement

**Figure 7:**
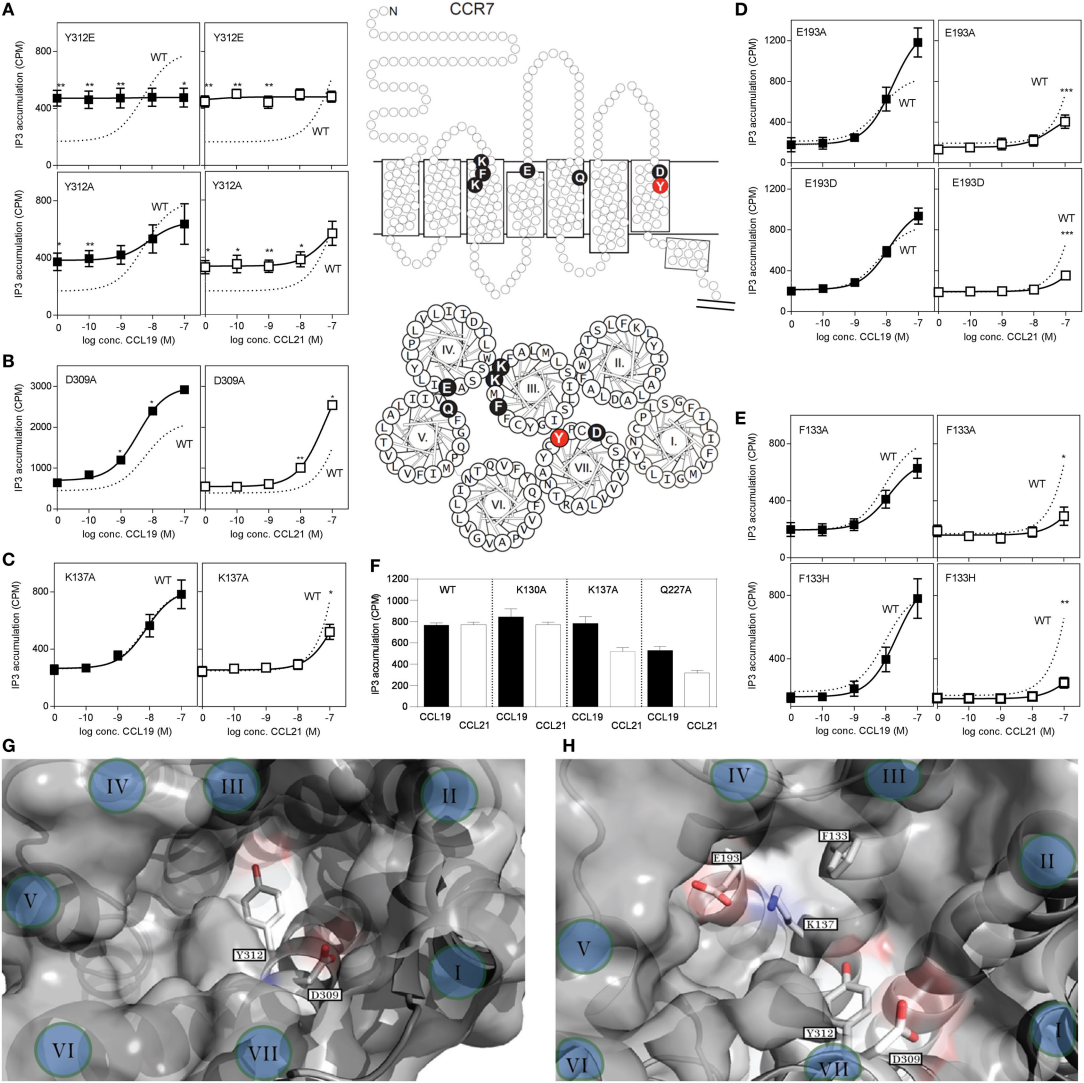
**Site-directed mutagenesis scan of CCR7**. **(A–E)** Dose–response curves of CCL19 (black squares) and CCL21 (white squares) on CCR7 WT (dotted line) and mutants obtained in PI-turnover assay measured in HEK293 cells cotransfected with CCR7 constructs and the chimeric G protein G_qi4myr_. **(A)** Mutational analyses of position 312 (Y312E, upper panel and Y312A, lower panel). **(B)** Mutation of position 309 (D309A) and **(C)** position 137 (K137A). **(D)** Mutation of position 193 (E193A, upper panel and E193D, lower panel). **(E)** Mutation of position 133 (F133A, upper panel and F133H, lower panel). **(F)** Effects of mutation K130A, K137A, and Q227A at 100 nM chemokine concentrations. Top **(G)** and side **(H)** view of the chemokine binding pocket of CCR7 based on homology-modeling from CCR5 (PDB reference 4MBS) (36) with helices indicated by roman letters. **(G)** Y312 is located at the border between the major (left) and minor (right) binding pocket, while D309 is located at the top of the minor binding pocket. A view into the major binding pocket presents the relative positions of F133, K137, and E193 **(H)**. Middle section: serpentine model, upper panel, and helical wheel, lower panel of CCR7 with indication of the included mutations. Statistical significance in signaling through WT CCR7 and mutants with either CCL19 or CCL21 was calculated using unpaired *t* test. **P < 0.01, *P < 0.05 (*n* = 3–9).

To investigate possible differences in the docking of CCL19 and CCL21 during CCR7 activation, we introduced mutations in CCR7 in the main binding pocket, in areas previously identified to be involved in ligand binding. The impact of single point mutations for CCL19- and CCL21-induced receptor activation was measured in PI-turnover in HEK293 cells transiently transfected with CCR7 and chimeric protein G_qi4myr_ (Figure 7). Most chemokine receptors carry a Glu in the top of TM7 (position VII:06/7.39) – a residue that function as an anchor for positively charged residues in chemokines as well as in small molecules (32). We use the generic numbering system suggested by Schwartz, followed by the Ballesteros−Weinstein numbering system (33–35). As the only endogenous chemokine receptor, CCR7 carries a Tyr at this position (at the border between the major and minor binding pocket), and thus this residue could be of selective importance for CCR7 interaction with its ligands. Introduction of Glu (Y312E) resulted in a very high degree of constitutive activity, corresponding to 50% of *E*_max_ for CCL19 on wt CCR7, and completely abolished ligand-induced activation (Figure 7A). A subsequent substitution to Ala (Y312A) uncovered that this Tyr is not needed for ligand-induced activation, as both ligands induced CCR7 activity with wt-like potencies [CCL19: EC_50_ of 5.4 nM (wt) and 7.8 nM (Y312A) and estimated CCL21: EC_50_ of 156 nM (wt) and 77 nM (Y312A)] (Figure 7A). Like for Y312E, Y312A induced constitutive activity in CCR7 to a basal activity of 30% of *E*_max_ for CCL19 on wt CCR7, demonstrating that the Tyr at position VII:06/7.39 restricts basal CCR7 activity – a property that ensures efficient migration toward a chemokine gradient.

Closer to the cell surface, helix 7 in CCR7 contains an acidic residue (Asp309^VII:03/7.36^) that could be envisioned to take over the usual role of Glu312^VII:06/7.39^ as chemokine recognition motif. Mutation of D309 to A (Figure 7B), revealed that Asp is not needed at this position for neither CCL19 nor CCL21 interaction with CCR7, in fact substitution with Ala increased the potency of both ligands, possibly due to removal of a negative charge that could repel the mildly acidic N-terminus of these ligands.

Mutation of K130^III:02/73.24^ to A, previously shown to affect both CCL19 and CCL21 signaling in CHO and COS-7 cells with up to 15-fold decreased potency (37), had no effect in our setup (Figure 7F). We used HEK293 cells and a different receptor construct (untagged CCR7 compared with C-terminally V5-his-tagged CCR7), possibly explaining this difference. Also in our model of CCR7, K130 points away from the binding pocket, and it is therefore not included in our pocket view model (Figures 7G,H).

Mutagenesis of K137^III:09/3.33^ to A in the bottom of the major binding pocket, sheltered under ECL2 of TM3 has previously been shown to selectively lead to diminished CCL21-induced G protein signaling through CCR7 with up to 22-fold decrease in potency of CCL21 compared with less than 2.5-fold decrease for CCL19, whereas changing Q227^V:08,5.42^ to A in the top of TM5 (same position on basolateral axis in major binding pocket, but further away from minor binding pocket than K137) affected both ligands with up to 20- and 28-fold decrease in potency for CCL19 and CCL21, respectively (37).

We confirm the data on K137A and Q227A (Figures 7C,F) and inferred that if this ligand selectivity reflects direct interaction differences, more residues in this receptor region would be similarly selective, and define a CCL21 recognition domain.

Focusing on the area of the major binding pocket delimited by TM3, -4, and -5, we mutated Glu193^IV:20/4.60^ – another putative negatively charged CCR7 anchor point for CCL19 and CCL21 (in the absence of any impact of Tyr312 and Asp 309 in TM7). Intriguingly, mutating E193 to A selectively impairs CCL21 signaling, whereas it has the opposite effect (enforcement of signaling) on CCL19 (Figure 7D). Even the most conservative substitution to Asp (E193D) impaired CCR7 activation by CCL21 with no impairment of CCL19, indicating that the length of the negatively charged side chain matters, and that we manipulated a finely tuned CCL21 interaction domain. How K137 and E193 affect CCL21 signaling is still open to interpretation; however, as they are positioned close to each other (Figure 7H), they likely affect CCL21 as a pair.

Having established that the top of TM3, -4, and -5 seems more important for CCL21 than for CCL19, primarily by focusing on charged residues, we looked into an aromatic residue (Phe133 in position III:05/3.29) in TM3, located between the two identified lysines (K130 and K137) and pointing right into the binding crevice. When F133 was mutated to Ala (F133A), this too resulted in a CCR7 receptor selectively unresponsive to CCL21 (Figure 7E). Even substitution with a histidine that has pseudo-aromatic properties, selectively interfered with CCL21 signaling. This residue, neither as Phe nor His, could be an interaction partner of K137 or E193, and accumulating experimental evidence suggests that this small region constitutes a part of a selective and *direct* CCL21 recognition domain. Finally, amino acids shown to be important for CCL21 activation, without affecting CCL19 induced signaling, was shown to be of equal importance to tailless CCL21, indicating that these variants of CCL21 probably activate the receptor in the same way (Table 1).

**Table 1:** **Tailless-CCL21 depends on the same amino acid interactions as CCL21 for G-protein signaling**.

	WT CCR7	K137A	E193A	F133A
CCL21	57.0 ± 0.7	16.0 ± 3.3	19.3 ± 4.6	28.7 ± 7.0
Tailless-CCL21	42.7 ± 2.0	16.7 ± 5.1	17.5 ± 3.4	27.1 ± 10.3

*Values annotate percentage activation of CCR7 (±SEM) in response to either CCL21 or tailless-CCL21 relative to CCL19*.

## Discussion

**Figure 8:**
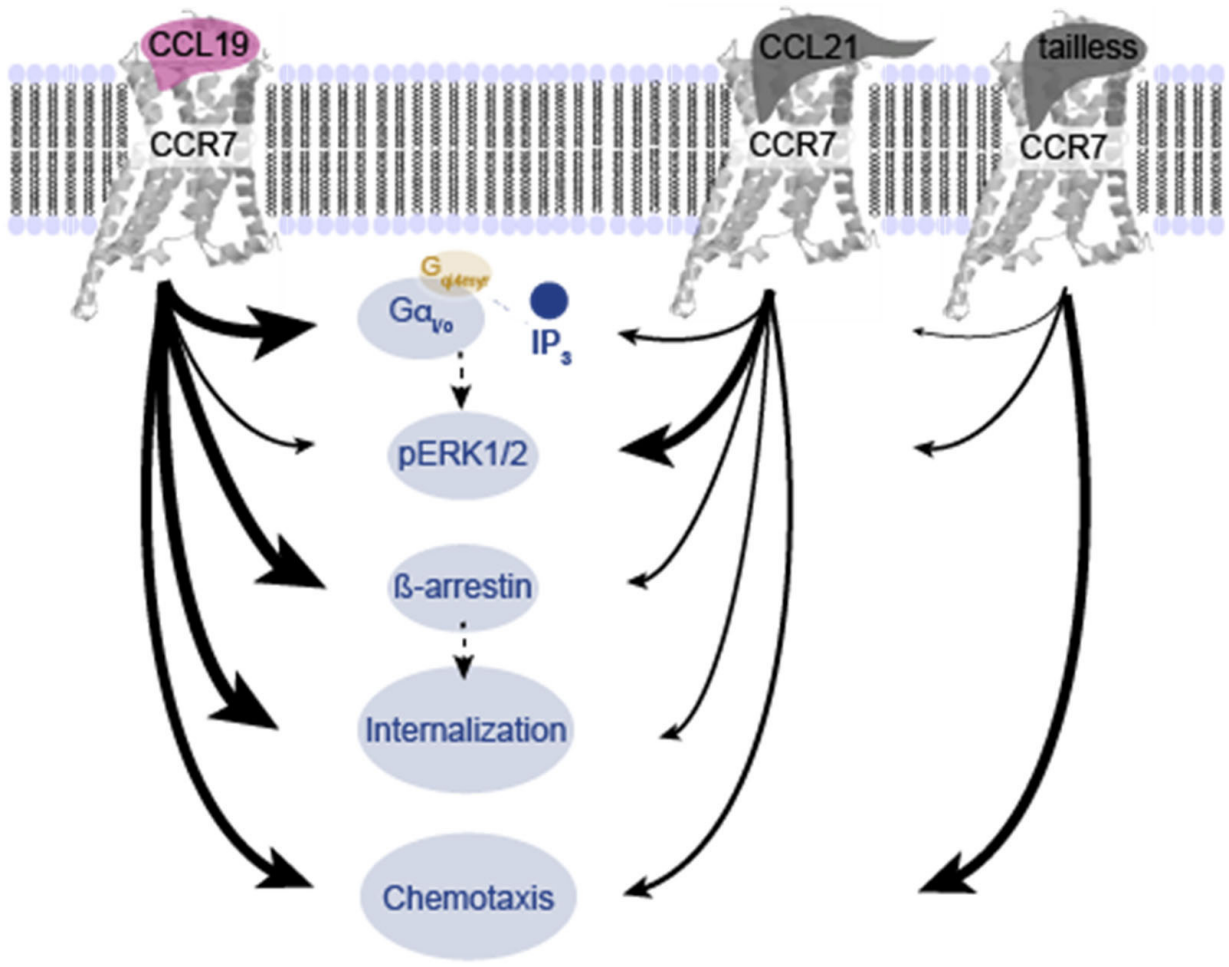
**Overview of differences in signaling induced by CCL19, CCL21, and tailless-CCL21**. Schematic illustration of the relative effect of CCL19, CCL21, and tailless-CCL21 on diverse cellular effects presented in Figures 1–6. Thickness of arrows signifies effect, i.e., thick arrow, high activity; thin arrow, low activity.

Bias is not uncommon in the chemokine system, as reviewed recently (18, 38). In addition to the present study in DCs and in experimental cell lines (summarized in Figure 8), ligand bias in CCR7 has been described in other cells (19–22). It has also been described for ligands acting on other endogenous [CXCR2, CXCR3, and CCR10 (18)] and viral chemokine receptors (39–41). Receptor bias has been described for instance in CCR5 wt and variants, where the same ligand elicited different signals in a receptor-dependent manner and is also common among virus-encoded receptors (42, 43). Tissue bias adds an extra dimension of complexity to the chemokine system with differential expression pattern in a spatial- and time-dependent manner for receptors and ligands (17, 18).

Investigating CCR7 bias, we chose to look at CCR7 in DCs that naturally express CCR7 and play a key role in both adaptive and acquired immunity. DC migration to lymph nodes dictated by CCR7 is of outmost importance for these processes. As the first, we show that CCL21 is less potent than CCL19 in inducing chemotaxis of human DCs. This contrasts earlier studies in murine DCs reporting CCL21 to be more potent compared with CCL19 in steep chemokine gradients, whereas no difference was found in shallow gradients (44). Also, murine DCs were shown to preferentially migrate toward CCL21, in opposing gradients of CCL19 and CCL21. In support of our study, another group described preferential migration of murine DCs toward CCL19 in a similar setup (15). In further support of our data, Ricart et al. found that in shallow gradients (≤ 20 nM source concentrations), CCL19-induced murine DC migration with a higher CI than CCL21, yet this was not the case at steep gradients (200 nM source concentrations) (45).

We show that tailless-CCL21 – a naturally occurring CCL21 product formed by protease activity in DCs (15) – is equally potent to CCL19 in inducing DC chemotaxis, which is in agreement with recent findings (14). It could be speculated that CCL21 accumulation on the DC surface through GAG binding generate a local reservoir that disturb DC gradient sensing. At very high CCL21 concentrations the gradient may become visible again supplying higher CCL21 concentrations than what can be accumulated on the DC surface. Our data on chemokine localization on the membrane shows that CCL21, but not tailless-CCL21 forms small and large puncta on the cell surface, consistent with an earlier study detecting CCL21 on the surface of cells transfected with CCR7 (46). That study showed that the formation of puncta depends on both CCR7 and GAGs, indicating that the spots are clusters of CCL21-bound CCR7, probably in combination with GAGs. This supports our theory that CCR7 on the surface of DCs exposed to CCL21 is covered in ligand and thus may be unable to sense weak CCL21 gradients. In contrast, in cells exposed to CCL19, CCR7/CCL19 complexes are quickly internalized and whereas the ligand is degraded, CCR7 is recycled to the membrane for renewed ligand engagement.

**Figure 9:**
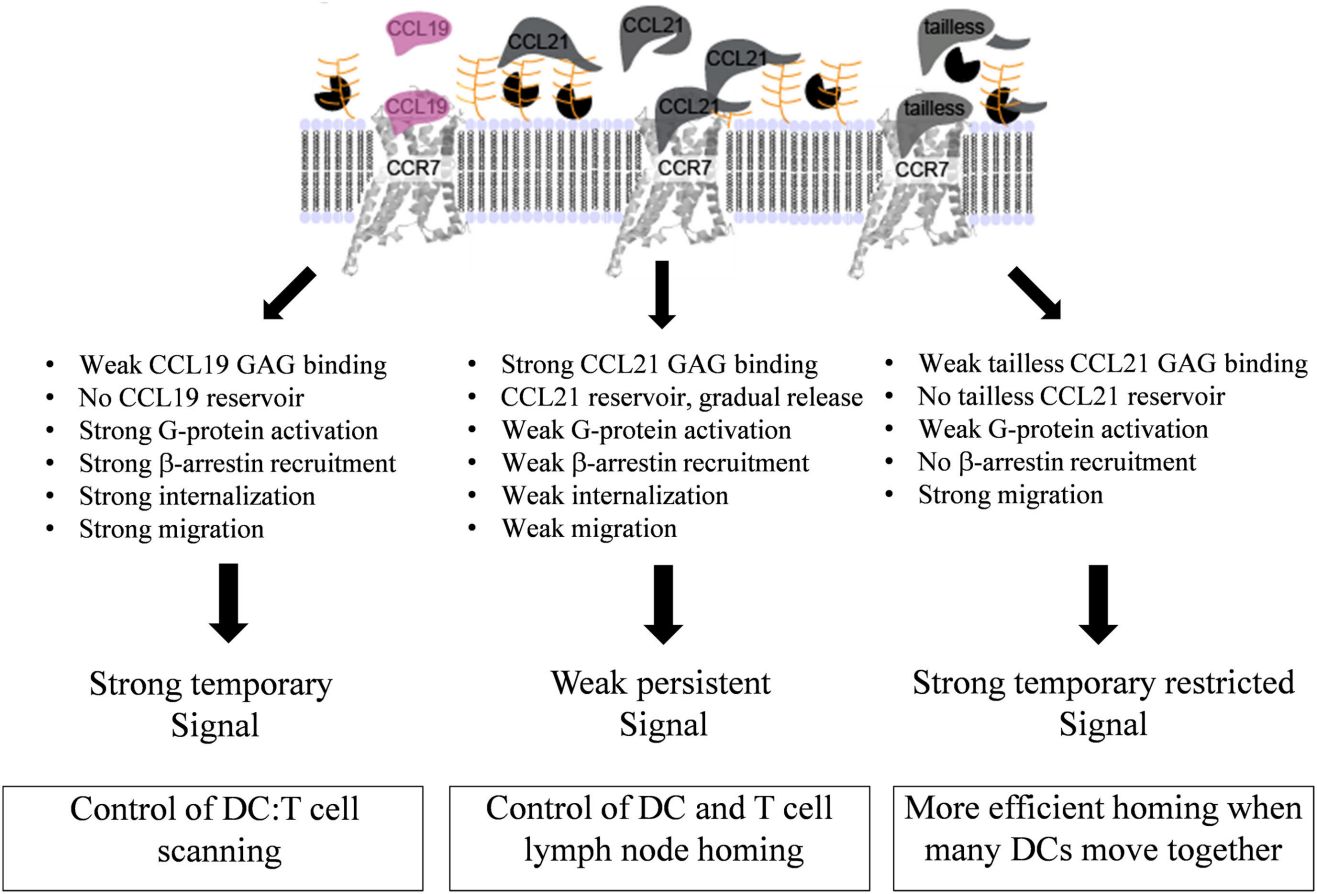
**Model for diverse mechanisms of CCL19, CCL21, and tailless-CCL21 in regulation of CCR7 activation**. As revealed by the structure comparison (Figure 5), CCL21 harbors structural motifs indicating a unique ability to strongly interact with GAGs, also supported by earlier reports. Increased GAG binding builds a local CCL21 reservoir at the cell surface, with CCL21 present in an inhibited, non-inhibited, or even facilitated form dependent on the GAG carrying it. In contrast, CCL19 only displays weak GAG interaction and readily diffuses away from the cell if not immediately bound to CCR7. GAG-bound CCL21 on the contrary, may either (i) interact directly with CCR7 if bound to polysialic acid, (ii) interact with CCR7 after GAG detachment caused by protease induced tail removal, with tailless-CCL21 probably being immediately ready for receptor engagement since release is expected to occur at the DC surface and thus in close proximity to CCR7 molecules ready to capture the chemokine, or (iii) interact with CCR7 after transfer from a GAG that presents CCL21 in its inhibited state (CS-B), to a GAG that allows CCR7 engagement (polysialic acid), with CS-B here acting as a dormant reservoir. CCL19 does not form a local reservoir, and once bound to CCR7, it gets quickly internalized and degraded, which is not the case for CCL21. Our model thus predicts that CCL19 and CCL21 induce differential CCR7 activation, with CCL19 creating a short-lived (temporary) signal, and CCL21 displaying a weaker, but more persistent CCR7 activation profile.

We speculate that haptotactic CCL21 gradients, earlier shown to gather peri-lymphatic and lymphatic capillary DC migration toward collecting lymphatic vessels (47), are converted *in situ* to short-lived tailless-CCL21 gradients of higher potency. Thereby, the truncated form of CCL21 could potentiate the chemotactic signal in cases where many activated DCs move together, with the front runners paving the way for the rest of the DCs, by leaving a trail of cleaved (tailless) CCL2 (Figure 9). This could increase DC homing in situations with massive DC activation, e.g., during a severe infection.

Since ERK activation is coupled to migration (48) we investigated CCL19- and CCL21-induced ERK activation in DCs. The observed more efficient and prolonged ERK1/2 activation by CCL21 compared with CCL19 is in contrast to earlier studies in HEK293 cells, where CCL19 displayed higher (19) or similar efficacy (21) as CCL21. These differences could be due to variations in GAG distribution in human DCs versus HEK293 cells, as we find that CCL21 interaction with polysialic acid is important for DC signaling revealed by the impact of NA treatment on CCL21-induced ERK1/2 activation. This treatment interferes with CCL21 signaling possibly by preventing a local CCL21 reservoir at the DC surface needed for higher and prolonged ERK activation (Figure 9). It may also alter CCL21 structure, and thereby function, by unlocking of an auto-inhibited CCL21, as described recently (14). In general, our data do not contradict that ERK activation could be an important player in directing CCR7-induced chemotaxis. At least one study reports that CCR7-G_i_ mediated activation of MAP kinases, including p38 and ERK1/2, in response to either CCL19 or CCL21, is important for DC chemotaxis, but not migrational speed, that was mainly controlled by Rho (49). On the other hand, PGE2, that is a key player in inducing migration of human monocyte-derived DCs (50, 51) and human monocytes, potently inhibits ERK1/2 activation in the latter (52). Along these lines it has been reported that ERK activation keeps human DCs in an immature state and that ERK inhibition by UO126 increases the expression of CCR7 and stimulates migration toward CCL19 (53). Similarly, human cord blood DCs experience improved migration toward CCL19 upon treatment with UO126 (54). In our hands, UO126 neither inhibited nor enhanced chemotaxis induced by either CCL19 or CCL21 (data not shown). A role for the Rho pathway in stimulation of murine DC chemotaxis *in vitro* and *in vivo* is supported by several studies (55, 56), and thus numerous pathways may be involved in controlling DC chemotaxis, and they may not be the same in 2D and 3D settings.

Many groups have studied signaling and internalization of CCR7. CCL19 and CCL21 have been reported to have the same efficacy and potency in G protein activation (in H9 T cell lymphoma cells) (19) and Ca^2+^ flux (in L1.2 cells, a murine pre-B cell line) (57). We observed an approximately sevenfold lower potency of CCL21 in inducing G protein activation compared with CCL19, and found that tailless-CCL21 behaved more like CCL21, although it seemed to have an even lower potency. This is in line with recent data reporting limited intracellular Ca^2+^ mobilization in response to a soluble version of CCL21 compared with CCL19 and CCL21 in human DCs (58).

Others have also shown that CCL19 is more potent than CCL21 in inducing CCR7 phosphorylation and β-arrestin 2 recruitment (19, 20). Consistent with earlier studies (19, 20, 22, 57), we observe weak CCR7 internalization in response to CCL21, whereas CCL19 induces strong β-arrestin 2-dependent internalization.

Importantly β-arrestin 1 and 2 coupling by CCR7 in response to both CCL19 and CCL21 seem to differentially affect ERK activation, with β-arrestin 1 coupling inhibiting ERK activation and β-arrestin 2 coupling stimulating ERK activation (21). That differential β-arrestin coupling by CCL19 and CCL21 dictates ligand bias is not likely, since differential β-arrestin-mediated phospho ERK is not observed between CCL19 and CCL21 (21).

Instead, differential recruitment of GRK molecules by CCR7 in response to CCL19 and CCL21 could be an important player in controlling ERK ligand bias, thus GRK2 and GRK3 recruitment by CCR7 in response to CCL19 negatively affects ERK activation, whereas these GRKs are not involved in controlling ERK activation induced by CCL21 (21). GRK6 knock down on the other hand was shown to potently inhibit ERK activation induced by both CCL19 and CCL21, and thus it seems the relative recruitment of GRK2, GRK3, and GRK6 is very important for determining ERK activation *via* CCL19, whereas the amount of GRK6 recruitment by CCR7 determines CCL21-induced ERK activation through this receptor.

As internalized CCR7 is recycled to the cell surface, while CCL19 is targeted for lysosomal degradation (20), it is conceivable, that CCL19-induced CCR7 endocytosis function as a negative feedback loop for CCL19, which is secreted by DCs (59), to prevent uncontrolled autocrine signaling. This may cause CCL19-mediated signaling to be temporal compared with that of CCL21 (Figure 9). Under normal circumstances, autocrine CCL19 is unlikely to influence DC lymph node homing, since CCL19 secretion from DCs is initiated after lymph node arrival (60). More likely, DC-secreted CCL19 serves as T-cell attractant controlling scanning behavior of naïve T-cells to increase cognate MHC-peptide encounter (61).

Our comprehensive structure comparison of CCL19 and CCL21 reveals that in addition to the elongated basic C-terminus, CCL21 contains a highly basic cluster of amino acids in the predicted GAG-binding area, not matched in CCL19 (Figure 6). This possibly adds to the unique ability of CCL21 to strongly interact with GAGs (12). Only CCL21, not CCL19, binds to chondroitin sulfate-B (CS-B) (62, 63), and whereas this binding inhibits CCL21-induced signaling, binding to heparan sulfate (HS) does not (62, 63). Similarly, the glycan polysialic acid was recently proven to act as important coreceptor for CCL21 (14). In contrast to CS-B and HS, polysialic acid is very restricted in its expression and apart from on neurons, where it regulates, e.g., migration, it is found mainly on leukocytes and DCs (14, 64, 65). The different roles of specific GAGs in regulating CCR7 activity, adds to the complexity of tissue bias. Our data suggests that the CCL21:GAG interaction is central for the different biological responses in DCs (Figure 9). Since mature DCs express a mixture of various GAGs on their surfaces, including CS-B, HS, and polysialic acid (65, 66), CCL21 captured on the surface of DCs may be presented in either an inhibited, non-inhibited, or even facilitated form dependent on the GAG carrying it. In addition, DC protease activity (15) likely alters the balance between free and GAG-coupled CCL21, potentially also influencing CCL21 activity. The GAG-tethered CCL21 on the DC surface may function as a local chemokine source that may (A) activate CCR7 directly or (B) become gradually available for receptor binding and activation by the DC to which it was tethered or DCs in the vicinity, either upon transfer between different GAG types or through gradually cleavage and release from inhibitory GAGs by endogenous proteases. Continuous release of CCL21 could potentially lead to a more continuous activation of CCR7, explaining the higher and prolonged ERK1/2 activation profile induced by this chemokine.

In summary, we present new data supporting a model in which CCL19- and CCL21-mediated CCR7 activation in DCs employ the same signaling pathways, but CCL19 is a temporal signal compared with CCL21, which is more persistent (Figure 9). This fits well with homing mediated *via* CCL21 as a prolonged process, whereas T-cell scanning mediated *via* CCL19 needs to be short-lived allowing for T-cells to quickly move around between different DCs. In addition we define tailless-CCL21 as a physiological relevant, functionally distinct chemokine probably equally important in controlling immune cell functions as CCL19 and CCL21. A central perspective of the present work is that since CCL19, CCL21, and tailless-CCL21 seem to activate CCR7 in different ways, it opens up the possibility of designing drugs that interfere with CCR7 signaling in a *ligand-specific* manner. CCR7 has been shown to drive cancer and to be involved in multiple sclerosis progression and development of graft-versus-host disease thus playing a dual role in health and disease (67–71). Biased drugs targeting specific ligands or signaling events of CCR7 could be a future solution in disease settings with aberrant CCR7 signaling. By targeting a subset of ligands or signaling paths, side effects caused by unwanted interference with beneficial CCR7 signaling important for normal immune responses in the patient could be avoided.

Future drugs designed to affect CCR7 signaling could target (1) the area between the minor and major binding pocket in this study represented by Y312 that upon mutation to either E or A confers massive constitutive activity to CCR7, identifying this area as an activity hot-spot, (2) the major binding pocket affecting mainly CCL21 receptor docking, thus enabling differentiation between CCL19 and CCL21, (3) GAG-binding obstructing CCL21-specific effects, without affecting tailless-CCL21 function, or finally (4) endogenous protease function or expression affecting only tailless-CCL21.

## Author Contributions

GH, OL, and AS wrote the manuscript, designed and carried out experiments, and analyzed and interpreted data. VD, CB, MH, SF, and SA contributed to data acquisition and/or data interpretation. MR wrote the manuscript, designed experiments, analyzed, and interpreted data. All the authors helped to revise, finally approved the manuscript, and agreed to be accountable for all aspects of the work.

## Conflict of Interest Statement

The authors declare that the research was conducted in the absence of any commercial or financial relationships that could be construed as a potential conflict of interest.

## References

[1] Viola A Luster AD. Chemokines and their receptors: drug targets in immunity and inflammation. Annu Rev Pharmacol Toxicol (2008) 48:171–97. 10.1146/annurev.pharmtox.48.121806.154841 17883327

[2] Yoshida R Imai T Hieshima K Kusuda J Baba M Kitaura M Molecular cloning of a novel human CC chemokine EBI1-ligand chemokine that is a specific functional ligand for EBI1, CCR7. J Biol Chem (1997) 272:13803–9. 10.1074/jbc.272.21.13803 9153236

[3] Bachelerie F Ben-Baruch A Burkhardt AM Combadiere C Farber JM Graham GJ International Union of Basic and Clinical Pharmacology. [corrected]. LXXXIX. Update on the extended family of chemokine receptors and introducing a new nomenclature for atypical chemokine receptors. Pharmacol Rev (2014) 66:1–79. 10.1124/pr.113.007724 24218476 PMC3880466

[4] Rot A von Andrian UH. Chemokines in innate and adaptive host defense: basic chemokinese grammar for immune cells. Annu Rev Immunol (2004) 22:891–928. 10.1146/annurev.immunol.22.012703.104543 15032599

[5] Forster R Davalos-Misslitz AC Rot A. CCR7 and its ligands: balancing immunity and tolerance. Nat Rev Immunol (2008) 8:362–71. 10.1038/nri2297 18379575

[6] Ueno T Saito F Gray DH Kuse S Hieshima K Nakano H CCR7 signals are essential for cortex-medulla migration of developing thymocytes. J Exp Med (2004) 200:493–505. 10.1084/jem.20040643 15302902 PMC2211934

[7] Misslitz A Pabst O Hintzen G Ohl L Kremmer E Petrie HT Thymic T cell development and progenitor localization depend on CCR7. J Exp Med (2004) 200:481–91. 10.1084/jem.20040383 15302903 PMC2211928

[8] Ohl L Mohaupt M Czeloth N Hintzen G Kiafard Z Zwirner J CCR7 governs skin dendritic cell migration under inflammatory and steady-state conditions. Immunity (2004) 21:279–88. 10.1016/j.immuni.2004.06.014 15308107

[9] Carlsen HS Haraldsen G Brandtzaeg P Baekkevold ES. Disparate lymphoid chemokine expression in mice and men: no evidence of CCL21 synthesis by human high endothelial venules. Blood (2005) 106:444–6. 10.1182/blood-2004-11-4353 15863780

[10] Randolph GJ Angeli V Swartz MA. Dendritic-cell trafficking to lymph nodes through lymphatic vessels. Nat Rev Immunol (2005) 5:617–28. 10.1038/nri1670 16056255

[11] Gunn MD. Chemokine mediated control of dendritic cell migration and function. Semin Immunol (2003) 15:271–6. 10.1016/j.smim.2003.08.004 15001176

[12] de Paz JL Moseman EA Noti C Polito L von Andrian UH Seeberger PH. Profiling heparin-chemokine interactions using synthetic tools. ACS Chem Biol (2007) 2:735–44. 10.1021/cb700159m 18030990 PMC2716178

[13] Patel DD Koopmann W Imai T Whichard LP Yoshie O Krangel MS. Chemokines have diverse abilities to form solid phase gradients. Clin Immunol (2001) 99:43–52. 10.1006/clim.2000.4997 11286540

[14] Kiermaier E Moussion C Veldkamp CT Gerardy-Schahn R de Vries I Williams LG Polysialylation controls dendritic cell trafficking by regulating chemokine recognition. Science (2016) 351:186–90. 10.1126/science.aad0512 26657283 PMC5583642

[15] Schumann K Lammermann T Bruckner M Legler DF Polleux J Spatz JP Immobilized chemokine fields and soluble chemokine gradients cooperatively shape migration patterns of dendritic cells. Immunity (2010) 32:703–13. 10.1016/j.immuni.2010.04.017 20471289

[16] Lefkowitz RJ Shenoy SK. Transduction of receptor signals by beta-arrestins. Science (2005) 308:512–7. 10.1126/science.1109237 15845844

[17] Kenakin T. The potential for selective pharmacological therapies through biased receptor signaling. BMC Pharmacol Toxicol (2012) 13:3. 10.1186/2050-6511-13-3 22947056 PMC3506267

[18] Steen A Larsen O Thiele S Rosenkilde MM. Biased and g protein-independent signaling of chemokine receptors. Front Immunol (2014) 5:277. 10.3389/fimmu.2014.00277 25002861 PMC4066200

[19] Kohout TA Nicholas SL Perry SJ Reinhart G Junger S Struthers RS. Differential desensitization, receptor phosphorylation, beta-arrestin recruitment, and ERK1/2 activation by the two endogenous ligands for the CC chemokine receptor 7. J Biol Chem (2004) 279:23214–22. 10.1074/jbc.M402125200 15054093

[20] Otero C Groettrup M Legler DF. Opposite fate of endocytosed CCR7 and its ligands: recycling versus degradation. J Immunol (2006) 177:2314–23. 10.4049/jimmunol.177.4.2314 16887992

[21] Zidar DA Violin JD Whalen EJ Lefkowitz RJ. Selective engagement of G protein coupled receptor kinases (GRKs) encodes distinct functions of biased ligands. Proc Natl Acad Sci U S A (2009) 106:9649–54. 10.1073/pnas.0904361106 19497875 PMC2689814

[22] Byers MA Calloway PA Shannon L Cunningham HD Smith S Li F Arrestin 3 mediates endocytosis of CCR7 following ligation of CCL19 but not CCL21. J Immunol (2008) 181:4723–32. 10.4049/jimmunol.181.7.4723 18802075 PMC7877961

[23] Heydorn A Ward RJ Jorgensen R Rosenkilde MM Frimurer TM Milligan G Identification of a novel site within G protein alpha subunits important for specificity of receptor-G protein interaction. Mol Pharmacol (2004) 66:250–9. 10.1124/mol.66.2.250 15266015

[24] Kostenis E Zeng FY Wess J. Functional characterization of a series of mutant G protein alphaq subunits displaying promiscuous receptor coupling properties. J Biol Chem (1998) 273:17886–92. 10.1074/jbc.273.28.17886 9651394

[25] Kissow H Hartmann B Holst JJ Viby NE Hansen LS Rosenkilde MM Glucagon-like peptide-1 (GLP-1) receptor agonism or DPP-4 inhibition does not accelerate neoplasia in carcinogen treated mice. Regul Pept (2012) 179:91–100. 10.1016/j.regpep.2012.08.016 22989472

[26] Graham FL van der Eb AJ A new technique for the assay of infectivity of human adenovirus 5 DNA. Virology (1973) 52:456–67. 10.1016/0042-6822(73)90341-3 4705382

[27] Granas C Lundholt BK Heydorn A Linde V Pedersen HC Krog-Jensen C High content screening for G protein-coupled receptors using cell-based protein translocation assays. Comb Chem High Throughput Screen (2005) 8:301–9. 10.2174/1386207054020741 16101006

[28] Love M Sandberg JL Ziarek JJ Gerarden KP Rode RR Jensen DR Solution structure of CCL21 and identification of a putative CCR7 binding site. Biochemistry (2012) 51:733–5. 10.1021/bi201601k 22221265 PMC3272885

[29] Veldkamp CT Kiermaier E Gabel-Eissens SJ Gillitzer ML Lippner DR DiSilvio FA Solution structure of CCL19 and identification of overlapping CCR7 and PSGL-1 binding sites. Biochemistry (2015) 54:4163–6. 10.1021/acs.biochem.5b00560 26115234 PMC4809050

[30] Allen SJ Crown SE Handel TM. Chemokine: receptor structure, interactions, and antagonism. Annu Rev Immunol (2007) 25:787–820. 10.1146/annurev.immunol.24.021605.090529 17291188

[31] Thiele S Rosenkilde MM Interaction of chemokines with their receptors – from initial chemokine binding to receptor activating steps. Curr Med Chem (2014) 21:3594–614. 10.2174/0929867321666140716093155 25039782

[32] Rosenkilde MM Schwartz TW GluVII:06 – a highly conserved and selective anchor point for non-peptide ligands in chemokine receptors. Curr Top Med Chem (2006) 6:1319–33. 10.2174/15680266106061319 16918451

[33] Schwartz TW. Locating ligand-binding sites in 7TM receptors by protein engineering. Curr Opin Biotechnol (1994) 5:434–44. 10.1016/0958-1669(94)90054-X 7765177

[34] Baldwin JM Schertler GF Unger VM. An alpha-carbon template for the transmembrane helices in the rhodopsin family of G-protein-coupled receptors. J Mol Biol (1997) 272:144–64. 10.1006/jmbi.1997.1240 9299344

[35] Ballesteros JA Weinstein H Integrated methods for the construction of three-dimensional models and computational probing of structure-function relations in G protein-coupled receptors. In: Sealfon SC, editor. Receptor Molecular Biology. New York: Academic Press (1995). p. 366–428.

[36] Tan Q Zhu Y Li J Chen Z Han GW Kufareva I Structure of the CCR5 chemokine receptor-HIV entry inhibitor maraviroc complex. Science (2013) 341:1387–90. 10.1126/science.1241475 24030490 PMC3819204

[37] Ott TR Pahuja A Nickolls SA Alleva DG Struthers RS. Identification of CC chemokine receptor 7 residues important for receptor activation. J Biol Chem (2004) 279:42383–92. 10.1074/jbc.M401097200 15284247

[38] Amarandi RM Hjorto GM Rosenkilde MM Karlshoj S. Probing biased signaling in chemokine receptors. Methods Enzymol (2016) 570:155–86. 10.1016/bs.mie.2015.09.001 26921946

[39] McLean KA Holst PJ Martini L Schwartz TW Rosenkilde MM. Similar activation of signal transduction pathways by the herpesvirus-encoded chemokine receptors US28 and ORF74. Virology (2004) 325:241–51. 10.1016/j.virol.2004.04.027 15246264

[40] Rosenkilde MM McLean KA Holst PJ Schwartz TW. The CXC chemokine receptor encoded by herpesvirus saimiri, ECRF3, shows ligand-regulated signaling through Gi, Gq, and G12/13 proteins but constitutive signaling only through Gi and G12/13 proteins. J Biol Chem (2004) 279:32524–33. 10.1074/jbc.M313392200 15155729

[41] Rosenkilde MM Schwartz TW. Potency of ligands correlates with affinity measured against agonist and inverse agonists but not against neutral ligand in constitutively active chemokine receptor. Mol Pharmacol (2000) 57:602–9. 10.1124/mol.57.3.602 10692502

[42] Steen A Sparre-Ulrich AH Thiele S Guo D Frimurer TM Rosenkilde MM. Gating function of isoleucine-116 in TM-3 (position III:16/3.40) for the activity state of the CC-chemokine receptor 5 (CCR5). Br J Pharmacol (2014) 171:1566–79. 10.1111/bph.12553 24328926 PMC3954493

[43] Steen A Thiele S Guo D Hansen LS Frimurer TM Rosenkilde MM. Biased and constitutive signaling in the CC-chemokine receptor CCR5 by manipulating the interface between transmembrane helices 6 and 7. J Biol Chem (2013) 288:12511–21. 10.1074/jbc.M112.449587 23493400 PMC3642299

[44] Haessler U Pisano M Wu M Swartz MA. Dendritic cell chemotaxis in 3D under defined chemokine gradients reveals differential response to ligands CCL21 and CCL19. Proc Natl Acad Sci U S A (2011) 108:5614–9. 10.1073/pnas.1014920108 21422278 PMC3078419

[45] Ricart BG John B Lee D Hunter CA Hammer DA. Dendritic cells distinguish individual chemokine signals through CCR7 and CXCR4. J Immunol (2011) 186:53–61. 10.4049/jimmunol.1002358 21106854

[46] Kawamura T Stephens B Qin L Yin X Dores MR Smith TH A general method for site specific fluorescent labeling of recombinant chemokines. PLoS One (2014) 9:e81454. 10.1371/journal.pone.0081454 24489642 PMC3904831

[47] Weber M Hauschild R Schwarz J Moussion C de V I Legler DF Interstitial dendritic cell guidance by haptotactic chemokine gradients. Science (2013) 339:328–32. 10.1126/science.1228456 23329049

[48] Huang C Jacobson K Schaller MD. MAP kinases and cell migration. J Cell Sci (2004) 117:4619–28. 10.1242/jcs.01481 15371522

[49] Riol-Blanco L Sanchez-Sanchez N Torres A Tejedor A Narumiya S Corbi AL The chemokine receptor CCR7 activates in dendritic cells two signaling modules that independently regulate chemotaxis and migratory speed. J Immunol (2005) 174:4070–80. 10.4049/jimmunol.174.7.4070 15778365

[50] Scandella E Men Y Gillessen S Forster R Groettrup M. Prostaglandin E2 is a key factor for CCR7 surface expression and migration of monocyte-derived dendritic cells. Blood (2002) 100:1354–61. 10.1182/blood-2001-11-0017 12149218

[51] Muthuswamy R Mueller-Berghaus J Haberkorn U Reinhart TA Schadendorf D Kalinski P. PGE(2) transiently enhances DC expression of CCR7 but inhibits the ability of DCs to produce CCL19 and attract naive T cells. Blood (2010) 116:1454–9. 10.1182/blood-2009-12-258038 20498301 PMC2938836

[52] Cote SC Pasvanis S Bounou S Dumais N. CCR7-specific migration to CCL19 and CCL21 is induced by PGE(2) stimulation in human monocytes: involvement of EP(2)/EP(4) receptors activation. Mol Immunol (2009) 46:2682–93. 10.1016/j.molimm.2008.08.269 19545899

[53] Aguilera-Montilla N Chamorro S Nieto C Sanchez-Cabo F Dopazo A Fernandez-Salguero PM Aryl hydrocarbon receptor contributes to the MEK/ERK-dependent maintenance of the immature state of human dendritic cells. Blood (2013) 121:e108–17. 10.1182/blood-2012-07-445106 23430108

[54] Li G Basu S Han MK Kim YJ Broxmeyer HE. Influence of ERK activation on decreased chemotaxis of mature human cord blood monocyte-derived dendritic cells to CCL19 and CXCL12. Blood (2007) 109:3173–6. 10.1182/blood-2006-04-014753 17179222 PMC1852231

[55] Vargas P Maiuri P Bretou M Saez PJ Pierobon P Maurin M Innate control of actin nucleation determines two distinct migration behaviours in dendritic cells. Nat Cell Biol (2016) 18:43–53. 10.1038/ncb3284 26641718 PMC5885286

[56] Tanizaki H Egawa G Inaba K Honda T Nakajima S Moniaga CS Rho-mDia1 pathway is required for adhesion, migration, and T-cell stimulation in dendritic cells. Blood (2010) 116:5875–84. 10.1182/blood-2010-01-264150 20881208

[57] Yoshida R Nagira M Kitaura M Imagawa N Imai T Yoshie O. Secondary lymphoid-tissue chemokine is a functional ligand for the CC chemokine receptor CCR7. J Biol Chem (1998) 273:7118–22. 10.1074/jbc.273.12.7118 9507024

[58] Hauser MA Kindinger I Laufer JM Spate AK Bucher D Vanes SL Distinct CCR7 glycosylation pattern shapes receptor signaling and endocytosis to modulate chemotactic responses. J Leukoc Biol (2016) 99:993–1007. 10.1189/jlb.2VMA0915-432RR 26819318

[59] Sanchez-Sanchez N Riol-Blanco L Rodriguez-Fernandez JL. The multiple personalities of the chemokine receptor CCR7 in dendritic cells. J Immunol (2006) 176:5153–9. 10.4049/jimmunol.176.9.5153 16621978

[60] Piqueras B Connolly J Freitas H Palucka AK Banchereau J. Upon viral exposure, myeloid and plasmacytoid dendritic cells produce 3 waves of distinct chemokines to recruit immune effectors. Blood (2006) 107:2613–8. 10.1182/blood-2005-07-2965 16317096 PMC1895384

[61] Kaiser A Donnadieu E Abastado JP Trautmann A Nardin A. CC chemokine ligand 19 secreted by mature dendritic cells increases naive T cell scanning behavior and their response to rare cognate antigen. J Immunol (2005) 175:2349–56. 10.4049/jimmunol.175.4.2349 16081805

[62] Hirose J Kawashima H Swope WM Springer TA Hasegawa H Yoshie O Chondroitin sulfate B exerts its inhibitory effect on secondary lymphoid tissue chemokine (SLC) by binding to the C-terminus of SLC. Biochim Biophys Acta (2002) 1571:219–24. 10.1016/S0304-4165(02)00232-5 12090936

[63] Hirose J Kawashima H Yoshie O Tashiro K Miyasaka M. Versican interacts with chemokines and modulates cellular responses. J Biol Chem (2001) 276:5228–34. 10.1074/jbc.M007542200 11083865

[64] Bruses JL Rutishauser U. Roles, regulation, and mechanism of polysialic acid function during neural development. Biochimie (2001) 83:635–43. 10.1016/S0300-9084(01)01293-7 11522392

[65] Rey-Gallardo A Escribano C Delgado-Martin C Rodriguez-Fernandez JL Gerardy-Schahn R Rutishauser U Polysialylated neuropilin-2 enhances human dendritic cell migration through the basic C-terminal region of CCL21. Glycobiology (2010) 20:1139–46. 10.1093/glycob/cwq078 20488940

[66] Wegrowski Y Milard AL Kotlarz G Toulmonde E Maquart FX Bernard J. Cell surface proteoglycan expression during maturation of human monocytes-derived dendritic cells and macrophages. Clin Exp Immunol (2006) 144:485–93. 10.1111/j.1365-2249.2006.03059.x 16734618 PMC1941969

[67] Shields JD I Kourtis C Tomei AA Roberts JM Swartz MA. Induction of lymphoidlike stroma and immune escape by tumors that express the chemokine CCL21. Science (2010) 328:749–52. 10.1126/science.1185837 20339029

[68] Takeuchi H Fujimoto A Tanaka M Yamano T Hsueh E Hoon DS. CCL21 chemokine regulates chemokine receptor CCR7 bearing malignant melanoma cells. Clin Cancer Res (2004) 10:2351–8. 10.1158/1078-0432.CCR-03-0195 15073111

[69] Coghill JM Carlson MJ Panoskaltsis-Mortari A West ML Burgents JE Blazar BR Separation of graft-versus-host disease from graft-versus-leukemia responses by targeting CC-chemokine receptor 7 on donor T cells. Blood (2010) 115:4914–22. 10.1182/blood-2009-08-239848 20185583 PMC2890182

[70] Kivisakk P Mahad DJ Callahan MK Sikora K Trebst C Tucky B Expression of CCR7 in multiple sclerosis: implications for CNS immunity. Ann Neurol (2004) 55:627–38. 10.1002/ana.20049 15122702

[71] Bielecki B Jatczak-Pawlik I Wolinski P Bednarek A Glabinski A. Central nervous system and peripheral expression of CCL19, CCL21 and their receptor CCR7 in experimental model of multiple sclerosis. Arch Immunol Ther Exp (Warsz) (2015) 63:367–76. 10.1002/ana.20049 25957582 PMC4572056

