# Corrigendum: Differential CCR7 Targeting in Dendritic Cells by Three Naturally Occurring CC-Chemokines

**DOI:** 10.3389/fimmu.2017.00089

**Published:** 2017-02-08

**Authors:** Gertrud M. Hjortø, Olav Larsen, Anne Steen, Viktorija Daugvilaite, Christian Berg, Suzan Fares, Morten Hansen, Simi Ali, Mette M. Rosenkilde

**Affiliations:** ^1^Faculty of Health and Medical Sciences, Department of Neuroscience and Pharmacology, The Panum Institute, University of Copenhagen, Copenhagen, Denmark; ^2^Department of Haematology, Center for Cancer Immune Therapy (CCIT), Copenhagen University Hospital, Herlev, Denmark; ^3^Medical Faculty, Institute of Cellular Medicine, Newcastle University, Newcastle upon Tyne, UK

**Keywords:** CCR7, CCL19, CCL21, tailless-CCL21, dendritic cell, biased signaling, ERK

**Funding**

This study was supported by the Danish Council for Independent Research, Medical Sciences, the Novo Nordisk Foundation, the Lundbeck Foundation, the A. P. Møller Foundation for the Advancement of Medical Science, the Aase and Einar Danielsen Foundation, the Hørslev Foundation, the Gangsted Foundation, Scleroseforeningen, Fonden for Neurologisk forskning, grant FP7-MC-ITN (POSAT, 606979), and the Carlsberg Foundation grant number 2013_01_0694 and CF14-0690.

**Clarification of Corrigendum:**

In the original article, we neglected to acknowledge the Carlsberg Foundation, grant number 2013_01_0694 and CF14-0690 to MR. The authors apologize for this oversight.

This error does not change the scientific conclusions of the article in any way.

## Conflict of Interest Statement

The authors declare that the research was conducted in the absence of any commercial or financial relationships that could be construed as a potential conflict of interest.

